# Assessing the yield and nutrient potential of horse gram mutants (*Macrotyloma uniflorum* Lam. Verdc.) an underutilized legume through a multi-environment-based experiment

**DOI:** 10.1038/s41598-024-67282-5

**Published:** 2024-07-15

**Authors:** Sumaiya Sulthana Jafarullakhan, Vaishnavi Vijayakumar, Kundan Veer Singh, Naaganoor Ananthan Saravanan, Veeranan Arun Giridhari, Sivakumar Rathinavelu, Balaji Kannan, Vanniarajan Chockalingam, Raveendran Muthurajan, Karthikeyan Subburamu, Selvaraju Kanagarajan, Sudhagar Rajaprakasam

**Affiliations:** 1 Centre for Plant Breeding and Genetics (CPBG), Tamil Nadu Agricultural University (TNAU), Coimbatore, India; 2 Sugarcane Research Station, TNAU, Melalathur, Vellore, India; 3 Department of Post Harvest Technology Centre, Agricultural Engineering College and Research Institute (AEC and RI), TNAU, Coimbatore, India; 4 Department of Crop Physiology, Crop Management Studies, TNAU, Coimbatore, India; 5 Department of Physical Sciences and Information Technology, AEC and RI, TNAU, Coimbatore, India; 6 Anbil Dharmalingam Agricultural College and Research Institute, TNAU, Trichy, India; 7 Directorate of Research, TNAU, Coimbatore, India; 8 Department of Plant Breeding, Swedish University of Agricultural Sciences, P.O. Box 190, 234 22 Lomma, Sweden

**Keywords:** Plant breeding, Plant genetics

## Abstract

The agronomic stability and nutritional importance of 30 (Test genotypes: 29 + Check: 1 = 30) promising horse gram mutants were evaluated in this multi-environment-based experiment (MEE). Attempts were made to (i) identify stable mutants for agronomic traits through AMMI and GGE biplot models, (ii) quantify nutritional traits, (iii) understand the linkage between yield and nutritional traits, and (iv) estimate physical (PP) and cooking properties (CP) of selected genotypes to fix their food-chain usability. The ANOVA of the pooled data exhibited significant differences among environments (E), genotypes (G), and GxE interaction. The combined AMMI and GGE results helped to identify a few good-yielding and stable genotypes (GYSM) (G1, G25, G3, and G27). The yield advantages of these GYSMs over the parent PAIYUR 2 are 42.99%, 34.63%, 28.68%, and 30.59% respectively. The nutrient profiling of mutants revealed (i) a significant coefficient of variation for macronutrients (fat: 29.98%; fibre: 20.72%, and protein: 5.01%), (ii) a good range of variation for micronutrients, and (iii) helped to identify macro (MaNSM) and micro nutrient-specific mutants (MiNSM). The relationship analysis between yield and nutrient traits ascertained that yield had (i) positivity with protein (r^2^ = 0.69) and negativity for micronutrients except for Mn (r^2^ = 0.63), Cu (r^2^ = 0.46), and B (r^2^ = 0.01) in GYSM, (ii) positivity with protein and fibre in MaNSM, and (iii) negativity with micronutrients in MiNSM. Of the GYSM, G1 and G25 offer scope for commercial exploitation, and their PP and CP analyses revealed that G1 can be used for pastry and baked product preparation while G25 for weaning foods. Cooking time exhibited positivity with seed size parameters and negativity with water absorption capacity (r^2^ = − 0.53). An LC–MS–MS-based amino acid (AA) fractionation study showed the effect of induced mutagenesis on the contents of amino acids and also revealed the significance of horse gram for its lysine and methionine contents.

## Introduction

Legumes play a critical role in global food security by providing essential macro and micronutrients^1^. It is considered an eco-friendly crop due to the reasons that (i) it does not require frequent irrigations and chemical fertilizers for its growth and productivity which avoids soil salinization and water table pollution respectively, (ii) it caters to its nitrogen requirements through its root nodules’ nitrogen-fixing ability, (iii) it enriches the soil organic carbon content because it is a deciduous crop which also supports the growth of the succeeding crop, and (iv) it can withstand the incidence of foliar diseases thereby avoids the use of plant protection chemicals. Its higher protein and nutrient content address dietary deficiencies, especially in regions with limited access to animal protein. Legumes also exhibit resilience to adverse climates, ensuring a stable food supply. Horse gram (*Macrotyloma uniflorum* Lam. Verdc.) is one such legume mostly cultivated under rain-fed situations in India. It is a functional food, has medicinal properties, and its ability to cure a variety of diseases is well documented^2^. It satisfies the nutritional requirements of humans and livestock. The nutrient composition of horse gram includes protein (18–29%), carbohydrate (57.2%), crude fiber (5.3%), minerals (3.2%) including calcium (287 mg/100 g), phosphorus (311 mg/100 g), and iron (8.4 mg/100 g)^3^. In southern states of India, because of photosensitivity, it is mainly cultivated during the *Rabi* season at the verge of completion of the north-west monsoon, covering 348.16 thousand ha with production and productivity of 226.21 thousand tonnes and 650 kg ha^−1^ respectively (www.indiastat.com). The untapped genetic yield potential in horse gram is attributed to reasons like photosensitivity, indeterminate growth habit, and non-synchronized maturity^4^. To ensure future nutritional security in the rainfed farming situations of India which accounts for approximately 60%, targeted research on plant breeding and biotechnology needs to be started and expanded^5^. As a part of this target, variability using mutation techniques was created for yield and growth habits in two popular horse gram varieties namely, PAIYUR 2 and CRIDA1-18R under a Board of Research in Nuclear Sciences (BRNS)- Government of India (GoI)-funded project. After testing the homozygous populations in various breeding cyclic experiments, 29 promising mutant genotypes were chosen for further exploitation based on their yielding potential and nutritional traits^6^. Stable performance and better adaptability are the main criteria used for selecting the ideal genotype(s) for variety release or germplasm registration. In a multiple environment-based-experiment (MEE), the phenotypic performances of test genotypes are greatly influenced by the variations in genotype (G), and climatic conditions (E). Estimating the real yielding potential and stability of genotypes in an MEE can be achieved by calculating the effects of G, E, and G x E. For this purpose, statistically robust models like AMMI and GGE can be employed^7^. The AMMI approach has proven effective in discriminating genotypes with stable performance over environments and is frequently used to analyze G x E interaction (GEI). GGE biplot is a modified method of AMMI^8^ and is effective in decomposing G and GEI compared with AMMI. The GGE biplot is also used to classify the mega environment, assess genotype rankings, and choose the discriminative and representative among the tested environments. The usefulness of AMMI and GGE has been well explained by several authors like Mahalingam et al.^9^ in mung bean, Rao et al.^10^ in pigeon pea, and Azam et al.^11^ in chickpea.

Generally, in crop improvement programs, the probabilities of evolving genotypes with double advantages (better yield and nutrient contents) are rare. Earlier, a positive relationship between increased yield and protein^12^ and a negative linkage between yield and quality^13^ were reported. Sudhagar et al.^1^ necessitated selecting the horse gram genotypes with a better-yielding potential and nutrient status for ensuring sustainable productivity and avoiding malnutrition in rainfed farming areas. The estimation of physical and cooking quality parameters needs to be included in the breeding program, that help a breeder select appropriate cultivars that satisfy grower and consumer preferences. An assessment of the (i) physical properties (PP) helps to achieve precision in mechanization from sowing to post-harvest processing^14^ and (ii) cooking quality properties (CP) decide the value addition. Further, for drawing meaningful inferences, the consistency of PP and CP has to be evaluated in an MEE^15^. Thus, in this MEE, the PP (size, bulk density (BD)), specific gravity (SG), germinability, and 1000 grain weight (TGW)) and CP (length and breadth elongation ratio (LER & BER)), cooking time (CT), cooking weight (CW), water absorption capacity (WA), and total soluble salts (TSS)) were estimated. The essential (EAA) and non-essential amino acid (NEAA) contents in legumes decide the protein value. Margier et al.^16^ reported the nutritional and bioactive profiles of legumes. Therefore, the current study focused both on breeder and nutritionist perspectives. From a breeder standpoint, it was aimed to (i) identify stable high-yielding horse gram mutants, and (ii) identify nutrient-specific genetic stocks through nutrient profiling. From a nutritionist’s/consumer’s view, attempts were made to (iii) understand the relationship between yield and quality, (iv) estimate the physical and cooking quality traits, and (v) assess the amino acid contents of selected mutants.

## Results and discussion

Horse gram is a life-supporting arid legume in rainfed areas of southern India whose yield potential can be improved by evolving variability for key quantitative and qualitative traits. Induced mutagenic techniques are effective in horse gram genome restructuring^5^ which eventually created considerable variability^6^. The breeding potency of this induced variability was assessed in the current experiment through an MEE-based stability analysis and nutrient profiling. The mean performance of horse gram mutants across three distinct environments for yield and its attributing traits are compared with parent PAIYUR 2 (Table 1). Horse gram is an indeterminate growth-type plant where increased plant height will lead to increased numbers of pod-bearing clusters. In the current experiment also, the mutants exhibited an increased height with shortened internodes (data is yet to be published) which resulted in a wider variation in the number of clusters per plant (69–247) and pods per plant (211–550). These are the main two characters that made the mutants excel in yield than the parent. Among the tested horse gram genotypes, G1 (1393.88 kg/ha) and G25 (1312.41 kg/ha) showed higher yield performance than the check PAIYUR 2 (974.79 kg/ha).

**Table 1:** Mean performance of horse gram genotypes for yield and its attributing traits (Average of three environments ± SE).

Genotype	DMY	NC	NP	NS	YPH	RANK
G1	123.52 ± 1.41	186.27 ± 2.64	550.93 ± 4.18	5.47 ± 0.15	1393.88 ± 20.81	1
G2	122.07 ± 0.77	106.59 ± 3.39	410.96 ± 31.66	4.89 ± 0.29	896.48 ± 71.49	16
G3	122.43 ± 0.51	106.17 ± 3.63	535.89 ± 24.95	5.44 ± 0.11	1254.39 ± 52.29	6
G4	124.16 ± 0.49	112.64 ± 2.13	365.59 ± 39.76	5.58 ± 0.15	918.85 ± 81.66	14
G5	120.58 ± 0.57	178.37 ± 32.02	381.44 ± 41.65	5.21 ± 0.12	815.75 ± 77.43	26
G6	119.31 ± 1.10	119.79 ± 0.90	442.52 ± 34.13	5.69 ± 0.09	971.19 ± 40.26	8
G7	125.27 ± 0.52	119.70 ± 0.62	364.21 ± 1.91	5.16 ± 0.09	945.19 ± 6.51	11
G8	121.35 ± 0.73	247.99 ± 1.11	536.07 ± 20.13	5.25 ± 0.30	1263.21 ± 41.86	5
G9	121.73 ± 1.15	118.92 ± 0.45	359.12 ± 2.29	4.70 ± 0.20	880.86 ± 25.72	19
G10	120.56 ± 0.29	150.88 ± 0.95	424.89 ± 12.19	4.44 ± 0.29	949.04 ± 37.55	10
G11	119.26 ± 1.28	101.59 ± 7.13	392.70 ± 22.47	5.48 ± 0.27	958.71 ± 65.45	9
G12	120.94 ± 0.59	110.26 ± 8.30	287.37 ± 3.69	5.11 ± 0.48	811.14 ± 44.88	29
G13	119.41 ± 0.58	122.62 ± 1.06	362.04 ± 0.42	4.99 ± 0.47	834.39 ± 67.29	24
G14	118.73 ± 1.15	125.78 ± 0.91	387.63 ± 3.82	5.35 ± 0.25	900.30 ± 27.84	15
G15	120.54 ± 0.62	157.71 ± 1.46	313.23 ± 1.54	5.09 ± 0.30	851.94 ± 34.81	23
G16	118.77 ± 0.55	102.97 ± 1.56	436.37 ± 20.75	4.35 ± 0.25	940.21 ± 54.28	13
G17	119.36 ± 1.27	132.17 ± 1.24	390.02 ± 5.28	4.26 ± 0.32	892.30 ± 42.82	17
G18	116.01 ± 0.47	177.05 ± 2.30	501.07 ± 22.77	4.31 ± 0.09	879.60 ± 42.66	20
G19	120.67 ± 0.88	128.25 ± 1.11	372.59 ± 45.87	4.63 ± 0.23	882.22 ± 104.37	18
G20	118.57 ± 0.66	201.70 ± 0.55	379.56 ± 25.33	4.46 ± 0.16	954.07 ± 60.65	21
G21	120.89 ± 0.48	102.59 ± 10.30	344.59 ± 56.97	5.79 ± 0.31	942.65 ± 100.40	12
G22	118.69 ± 0.53	152.58 ± 28.20	546.44 ± 37.01	5.33 ± 0.38	1291.53 ± 34.02	3
G23	122.48 ± 0.33	100.50 ± 20.72	299.83 ± 2.82	5.25 ± 0.37	770.20 ± 34.00	30
G24	120.68 ± 0.64	164.89 ± 0.58	331.57 ± 2.70	5.00 ± 0.00	812.50 ± 11.67	27
G25	108.89 ± 1.06	124.81 ± 10.97	532.67 ± 10.84	5.78 ± 0.11	1312.41 ± 10.65	2
G26	119.78 ± 0.62	146.80 ± 1.08	529.07 ± 47.22	4.58 ± 0.04	859.39 ± 38.30	21
G27	118.86 ± 0.48	119.76 ± 16.64	518.89 ± 43.90	5.44 ± 0.29	1272.95 ± 30.31	4
G28	122.30 ± 0.67	137.78 ± 14.40	306.42 ± 2.25	5.19 ± 0.24	811.27 ± 39.91	28
G29	129.00 ± 2.12	156.22 ± 1.31	401.22 ± 8.52	4.78 ± 0.11	820.26 ± 22.44	25
G30	127.04 ± 0.49	69.35 ± 1.35	211.24 ± 1.47	3.00 ± 0.00	974.79 ± 1.21	7

*DMY* Days to maturity, *NC* Number of clusters per plant, *NP* Number of pods per plant, *NS* Number of seeds per pod, *YPH* Yield per hectare, *SE* Standard error.

The study of variation among the genotypes is imperative to realize the influence of environment on the crop growth^9^. The pooled ANOVA results (Table 2.) showed significant differences among G, E, and GEI. This indicates the presence of environmental influence on the trait expression. Rao et al.^10^ also showed similar results for grain yield in pigeon pea genotypes.

**Table 2:** Multiple-environment-based AMMI analysis derived ANOVA for yield and its related traits in horse gram genotypes.

Source	df	MSS				
		DMY	NC	NP	NS	YPH
Environments (E)	2	84.90**	2375.92**	23,268.44**	1.13	85,573.59*
Genotypes (G)	29	108.88**	12,064.13**	71,987.50**	3.08**	286,526.09**
Interactions (G X E)	58	3.98	892.87**	5386.14**	0.49**	21,113.98**
PCA1	30	6.29	1440.68**	6647.82**	0.75**	27,867.01**
PCA2	28	1.50	305.93**	4034.33**	0.21	13,878.59**
Residuals	174	5.69	19.97	100.35	0.31	5304.27

*DMY* Days to maturity, *NC* Number of clusters per plant, *NP* Number of pods per plant, *NS* Number of seeds per pod, *YPH* Yield per hectare, *df* degree of freedom, *MSS* Mean sum of square.

**Significance at 1% level, * significance at 5% level.

## Stability analysis

### *AMMI* biplot analyses

AMMI biplots are graphical representations of results and are used to gain insights into GEI on a specific trait expression. AMMI 1 biplots are generated based on the first principal component (PC1) and are used to interpret the mean performance and stability of genotypes over environments^17^. The vertical axis in an AMMI 1 biplot denotes PC1 values, suggesting that genotypes or environments positioned closely along a vertical line tend to have similar mean values. While the horizontal axis represents the main effects (G or E), genotypes aligned along this axis have similar interaction patterns^17^.

**Figure 1:**
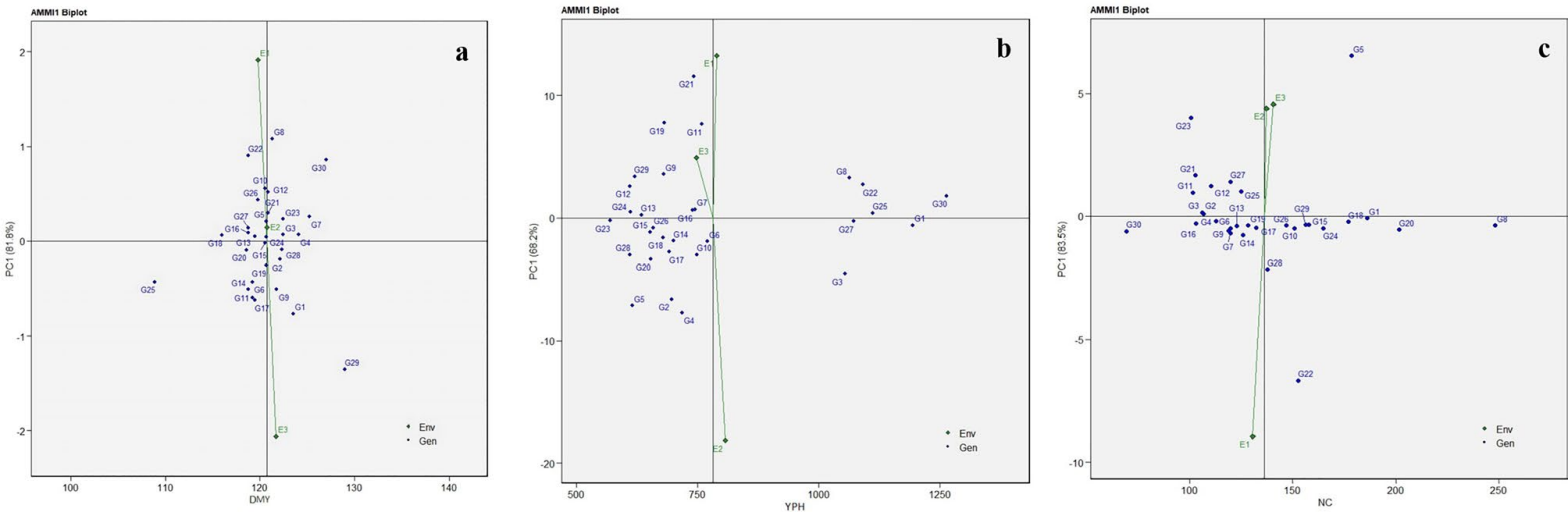
The AMMI 1 biplot shows the main effect and Principal Component (PC) 1 of both genotypes and environments of 30 horse gram genotypes in three different locations for the traits days to maturity -DMY (**a**), yield per hectare-YPH (**b**), and number of clusters per plant -NC (**c**).

The PC1 scores for both G and E were plotted for DMY, NC, NP, NS, and YPH. The PC1 scores for these traits are 81.8%, 83.5%, 63.8%, 79.6%, and 68.2% respectively. In the AMMI 1 biplot for the trait DMY (Fig. 1a), the genotypes G25, G11, G6, G14, and G20 expressed lower main effects indicating that these genotypes had a lower mean value but they showed relatively consistent performance across the study environments. These genotypes assume importance in drought avoidance breeding programs by virtue of lower maturity duration. Developing early maturing crop varieties has been a good breeding strategy for crops grown under drought^18^. The genotypes G3, G27, G1, G25, G22, and G8 exhibited higher main effects for YPH revealing their superior ability to adapt to different environments (Fig. 1b). The environments E1 and E2 expressed higher main effects for the trait YPH, indicating their productiveness for many genotypes (Fig. 1b). The genotypes G22, G28, G10, G18, G1, G8, G5, G6, G3, G27, and G27 are promising for NC, NP, and NS respectively (Fig. 1c) and (Supplementary Fig. S2a,b). Similarly, Mwiinga et al.^19^ classified G and E based on main and interaction effects in soybean.

In the AMMI 2 biplot, both PC1 and PC2 scores are used to elucidate the intricacies of GEI across multiple environments and help to identify better-adapting genotypes^7^. Genotypes placed near to origin in the biplot are considered more stable. For DMY, the genotypes G3, G4, G6, and G20 are close to the origin (Supplementary Fig S1a). For YPH (Supplementary Fig S1b), the genotypes G1, G27, G25, and G7 are nearer to the origin and therefore tagged as stable performers. Alike results for YPH are reported in pigeon pea by Rao et al.^10^. Genotypes G2, G3, and G18 are closer to the origin indicating that they are stable and ideal genotypes for the trait NC (Supplementary Fig S1c). Similarly, for the trait NP (Supplementary Fig. S3a), the genotypes G25, G10, G30, and G17 and for NS (Supplementary Fig. S3b), G18, G1, G21, G26, and G5 are marked as stable genotypes.

Combined results of the AMMI 1 and AMMI 2 biplot reveal that genotypes G1, G25, and G27 consistently demonstrated higher mean effects (significant adaptability) in AMMI 1, indicating their superior adaptability to different environments for the trait YPH. Additionally, the nearness of these genotypes to the origin in AMMI 2 biplots, demonstrates their performance reliability.

### *AMMI* based indices

#### *AMMI*-based statistics- Genotype Stability Index (GSI)

The ASV (AMMI Stability Value) and mean of a trait are used to rank the genotypes. The genotypes were ranked based on the lowest ASV and the highest mean for a trait. Consequently, these ranks are combined to arrive at a single comprehensive selection index GSI^20^ for the trait. The genotypes with the lowest GSI are considered stable genotypes. The ASV and GSI scores of thirty horse gram genotypes across three environments are furnished in Table 3. For the trait DMY, G4, G3, G28, G7, and G23 are identified as stable genotypes. These genotypes shall be considered for evolving variability for maturity duration. Similarly, for YPH, G1, G27, G7, and G16 are tagged as suitable performers and offer the scope of potential concerning horse gram yield enhancement programs. Additionally, G1, G8, and G3 exhibited lower GSI values for the other traits, namely NC, NP, and NS. However, these genotypes though possessed good index scores for yield-related traits but failed to achieve a higher mean value for yield. Typically, the indeterminate growth pattern of horse gram usually leads to an increased number of clusters and pods, resulting in higher yields. The correlation between these traits and yield was also reported in mung beans by Singh et al.^21^. However, variations in temperature, soil moisture conditions, dew, and other climatic elements could impact pod development and seed setting, contributing to the observed discrepancies in yield outcomes among these genotypes. Earlier, in soybeans, Hailemariam Habtegebriel^22^ identified stable genotypes using GSI values.

**Table 3:** Estimates of stability parameters of horse gram genotypes across three environments.

	DMY					NC					NP					NS					YPH				
	Mean	ASV	RA	RM	GSI	Mean	ASV	RA	RM	GSI	Mean	ASV	RA	RM	GSI	Mean	ASV	RA	RM	GSI	Mean	ASV	RA	RM	GSI
G1	123.51	3.31	26	5	31	186.27	0.38	1	3	4	550.92	2.38	13	1	14	5.46	0.44	11	5	16	1393.88	1.24	1	1	2
G2	122.07	0.78	11	9	20	106.59	0.45	2	24	26	410.96	7.45	25	12	37	4.88	1.55	26	20	46	896.48	6.61	16	16	32
G3	122.43	0.22	1	7	8	106.16	0.78	3	25	28	535.88	6.27	22	4	26	5.43	0.75	16	8	24	1254.39	17.07	28	6	34
G4	124.16	0.25	2	4	6	112.64	1.05	4	22	26	365.59	10.37	28	20	48	5.58	0.95	21	2	23	918.85	6.68	17	14	31
G5	120.58	1.27	15	16	31	178.37	32.99	29	4	33	381.44	10.82	29	17	46	5.20	0.79	18	13	31	815.75	6.73	18	26	44
G6	119.30	2.10	19	22	41	119.79	2.40	13	18	31	442.51	5.51	19	9	28	5.57	0.95	22	3	25	971.19	4.92	12	8	20
G7	125.27	1.22	14	3	17	119.69	3.52	20	20	40	364.20	1.80	5	21	26	5.16	0.22	5	15	20	945.19	2.96	6	11	17
G8	121.34	4.94	29	11	40	247.99	1.97	10	1	11	536.07	5.91	20	3	23	5.45	0.29	9	6	15	1263.21	5.25	13	5	18
G9	121.72	2.26	20	10	30	118.91	2.92	18	21	39	359.12	2.05	8	23	31	4.70	0.48	12	22	34	880.86	6.03	14	19	33
G10	120.55	2.50	23	17	40	150.87	2.49	15	10	25	424.88	2.55	14	11	25	4.44	0.68	15	26	41	949.04	4.25	10	10	20
G11	119.25	2.81	25	23	48	101.59	4.87	22	28	50	392.70	6.67	23	14	37	5.48	0.96	23	4	27	958.71	18.97	29	9	38
G12	120.93	2.39	22	12	34	110.25	6.19	24	23	47	287.37	2.28	11	29	40	5.11	2.00	29	16	45	811.14	14.24	25	29	54
G13	119.40	0.57	9	20	29	122.61	2.08	12	17	29	362.03	1.71	4	22	26	4.98	1.99	28	19	47	834.39	8.11	20	24	44
G14	118.72	2.26	21	26	47	125.77	3.86	21	15	36	387.62	2.30	12	16	28	5.34	0.54	13	10	23	900.30	25.00	30	15	45
G15	120.54	0.28	3	18	21	157.70	1.74	7	7	14	313.23	1.89	6	26	32	5.08	1.05	24	17	41	851.94	8.22	21	23	44
G16	118.76	0.40	7	25	32	102.96	1.55	6	26	32	436.37	5.35	18	10	28	4.34	0.54	14	27	41	940.21	2.23	4	13	17
G17	119.35	2.67	24	21	45	132.16	2.45	14	13	27	390.02	1.09	2	15	17	4.25	0.91	20	29	49	892.30	3.45	9	17	26
G18	116.01	0.38	6	29	35	177.04	1.20	5	5	10	501.07	3.34	16	8	24	4.30	0.06	1	28	29	879.60	3.22	8	20	28
G19	120.66	1.18	13	15	28	128.24	1.96	9	14	23	372.59	8.75	27	19	46	4.62	0.83	19	23	42	882.22	3.00	7	18	25
G20	118.56	0.37	4	28	32	201.70	2.78	17	2	19	379.55	3.80	17	18	35	4.45	0.21	3	25	28	854.07	1.74	2	21	23
G21	120.88	1.43	16	13	29	102.59	8.39	26	27	53	344.59	15.08	30	24	54	5.44	0.21	4	7	11	942.65	6.41	15	12	27
G22	118.69	4.20	28	27	55	152.58	33.79	30	9	39	546.44	6.67	24	2	26	5.33	2.04	30	11	41	1291.53	7.56	19	3	22
G23	122.48	0.91	12	6	18	100.50	20.22	28	29	57	299.82	2.15	10	28	38	5.24	1.42	25	12	37	770.20	9.74	24	30	54
G24	120.67	0.62	10	14	24	164.80	2.57	16	6	22	331.56	2.13	9	25	34	5.00	0.26	6	18	24	812.50	2.91	5	27	32
G25	108.88	1.94	17	30	47	124.81	5.64	23	16	39	532.66	0.83	1	5	6	5.77	0.75	17	1	18	1312.41	16.73	27	2	29
G26	119.77	2.09	18	19	37	146.80	1.99	11	11	22	529.07	7.75	26	6	32	4.58	0.14	2	24	26	859.39	15.29	26	21	47
G27	118.86	0.52	8	24	32	119.75	8.32	25	19	44	518.88	6.25	21	7	28	5.42	1.63	27	9	36	1272.95	4.51	11	4	15
G28	122.29	0.37	5	8	13	137.77	11.74	27	12	39	306.48	1.96	7	27	34	5.18	0.37	10	14	24	811.27	1.93	3	28	31
G29	129.00	6.07	30	1	31	156.22	1.75	8	8	16	401.22	3.28	15	13	28	4.77	0.28	8	21	29	820.26	9.63	23	25	48
G30	127.04	3.76	27	2	29	69.35	3.10	19	30	49	211.24	1.40	3	30	33	3.00	0.26	7	30	37	974.79	8.29	22	7	29

*DMY* Days to maturity, *NC* Number of clusters per plant, *NP* Number of pods per plant, *NS* Number of seeds per pod, *YPH* Yield per hectare, *ASV* AMMI stability value, *RA* Rank of the ASV, *RM* Rank of trait mean, *GSI* Genotype selection index.

### GGE biplot analyses

#### Discriminativeness versus representativeness biplot analysis

**Figure 2:**
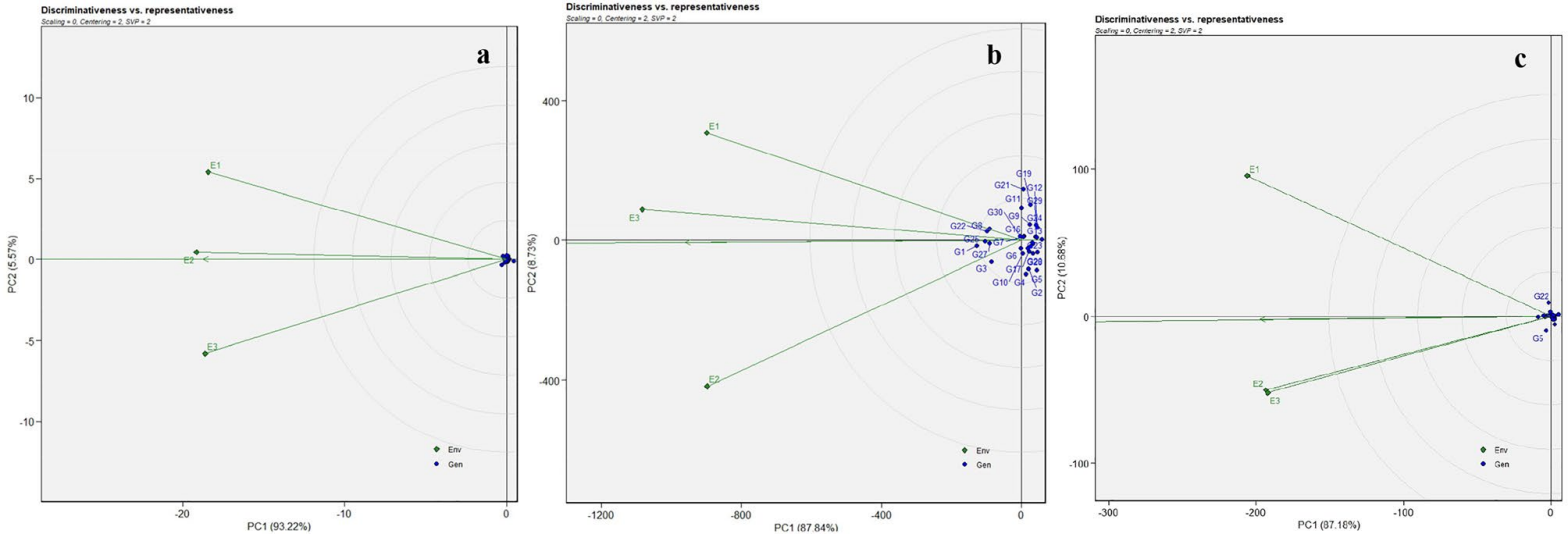
Discriminativeness vs. representativeness pattern of 30 horse gram genotypes in three different locations for the traits days to maturity (**a**), yield per hectare (**b**), and number of clusters per plant (**c**).

GGE biplots are a valuable tool for assessing the GEI, identifying mega-environments (ME), and discerning superior genotypes. In GGE biplot analyses, for identifying the MEs, ranking genotypes, and determining stable environments, scatter plots are utilized^23^. The discriminativeness vs. representativeness biplot illustrates the effectiveness of an environment in genotype discrimination. In the current experiment, Fig. 2a,b,c and (Supplementary Fig. S4a,b) illustrate the discriminativeness *vs*. representativeness of GGE analysis.

It is inferred that environment E3 stands out with its longest vector suggesting its robust ability to differentiate genotypes, and is considered as the best environment for the expression of traits DMY, YPH, and NS. Likewise, E1 is effective at distinguishing the genotypes for the traits NC and NP. Environment E2 had the shortest vector for the traits NC, NS, and YPH indicating all the genotypes performed equally in this environment. Similar findings based on discriminativeness *vs.* representativeness were made in soybeans by Natraj et al.^24^.

#### Mean versus stability biplot analysis

**Figure 3:**
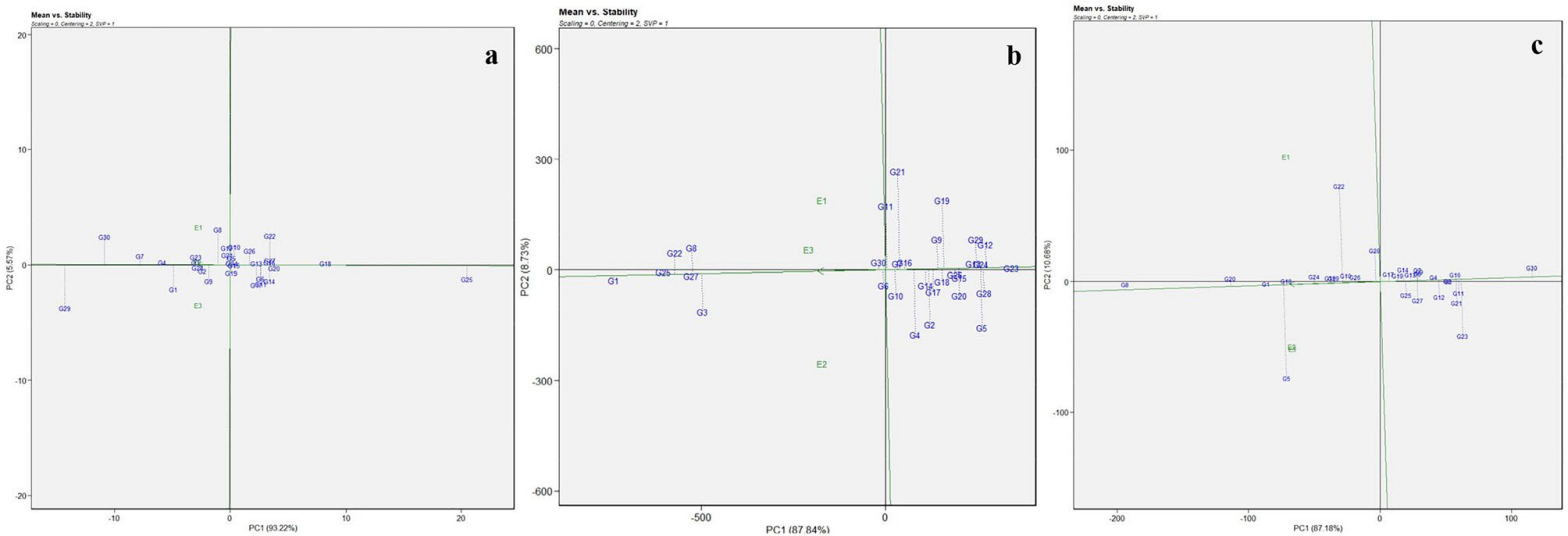
Mean vs. stability plots for 30 horse gram genotypes in three different locations for the traits days to maturity (**a**), yield per hectare (**b**), and number of clusters per plant (**c**).

The mean performance of the genotypes over environments can be interpreted from mean *vs.* stability biplots. Genotype stability can be assessed through the average environment coordinate (AEC) which passes through the origin. A genotype is considered stable when it forms a short and vertical line with the AEC axis^25^. For the trait, DMY, (Fig. 3a), G18, G16, G13, and G20 are located in close proximity to the AEC. The genotypes G1, G27, and G25 are positioned adjacent to the AEC and are recognized as both stable and highly productive for the trait YPH (Fig. 3b). The genotypes G16, G4, G8, and G1 for the trait NC (Fig. 3c); G7, G24, G12, and G1 for the trait NP (Supplementary Fig. S5a); and G29, G9, G1, and G7 for the trait NS (Supplementary Fig. S5b) are found stable and productive. Similarly, ideal stable lines were identified by Kumar et al.^26^ based on mean *vs.* stability biplots.

#### Which-won -where biplot

**Figure 4:**
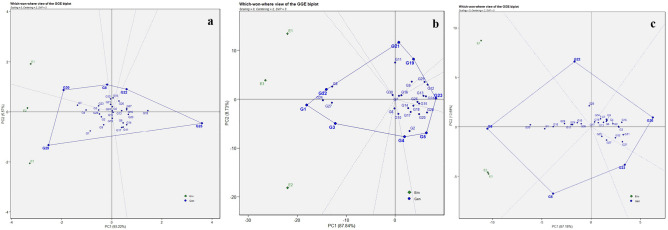
“Which-won-where” plots for 30 horse gram genotypes in three different locations for the traits days to maturity (**a**), yield per hectare (**b**), and number of clusters per plant (**c**).

The ‘Which-won-where’ plot serves as a valuable tool for analyzing the performance of a genotype in different environments. In this biplot, the polygon is created by linking the outermost genotypes from the biplot’s center. Perpendicular lines divide this polygon into sections, aiding in the visualization of ME. The top-performing genotypes are positioned at the vertices^22^. For the trait DMY (Fig. 4a), the environments are grouped into two MEs. ME1 consisting of E1 whereas, E2 and E3 forms ME2. G30 and G29 are positioned at the vertices of these respective mega-environments, indicating their better performance. For the trait YPH (Fig. 4b), all three environments are grouped as one ME where, G3, G1, and G22 are located at the vertex indicating that they are promising genotypes for YPH. Similarly, for NC (Fig. 4c), a single ME emerged, and within it, G8 stands out as the superior genotype. G22 and G1 are at the vertices of ME1 (E1 and E3) whereas G3 is in the ME2 (E2) for the trait NP (Supplementary Fig. S6a). For NS, (Supplementary Fig. S6b), three distinct MEs were formed. ME1 did not have genotypes at its vertex. In ME2, G25 holds the vertex position, while in ME3, G22 occupies the vertex.

From the summarized results of GGE biplots, it is inferred that the genotypes G1, G25, G3, and G27 are identified as stable and high-yielding genotypes whose yield potential is also higher than the parent PAIYUR 2. This stability can be advantageous for agricultural practices, as it provides a more predictable outcome regardless of varying environmental conditions.

Through a comprehensive examination of AMMI and GGE biplot analysis outcomes, the genotypes G1, G3, G25, and G27 have been identified as stable, indicating that they are less sensitive to environmental variations, making them desirable candidates and thus can be considered for further commercial exploitation either as a donor for yield improvement programs or can directly be released as a variety(ies).

#### Nutrient potential

Horse gram seeds are a rich source of carbohydrates, protein, amino acids, phosphorus, molybdenum, iron, and vitamins^1,2^. The nutrient potential (macro and micronutrients) of test genotypes was analyzed across three distinct environments, pooled, and overall mean values are considered for interpretation (Table 4). The relative variability for a nutrient trait can be realized through the coefficient of variation (CV). For the macronutrient, a maximum CV is observed for fat content (29.98%) followed by fibre (20.72%), and protein (5.01%). The observed variation in the macronutrients can be attributed predominantly to the effects of induced mutagenesis. Among the studied genotypes, G3 (25.44%) had the highest protein content, followed by G22 (24.98%) and G1 (23.91%). G19 (2.36%) showed the highest fat content. The genotypes G8 (2.82%) and G27 (2.57%) recorded higher fibre content than the parent PAIYUR 2 (1.84%). Similar investigations regarding macro-nutrients in horse gram have been reported by Sudhagar et al.^1^, Marimuthu and Krishnamoorthi^27^, and Vaishnavi et al.^28^. These genotypes can serve as potential nutritional stock for horse gram breeding programs aiming for macronutrient improvement.

**Table 4:** Nutritional potential of 30 horse gram genotypes across 3 different environments (Average of three environments ± SE).

	Macronutrients (%)			Micronutrients (mg/100 g)									
	Protein	Fat	Fibre	B	Mg	P	K	Ca	Mn	Fe	Cu	Zn	Mo
G1	23.91 ± 0.11	2.34 ± 0.21	1.60 ± 0.32	5.96 ± 0.05	167.93 ± 1.19	308.55 ± 3.69	1053.49 ± 1.64	290.18 ± 1.62	9.56 ± 0.38	2.62 ± 0.33	2.30 ± 0.66	4.83 ± 0.57	0.25 ± 0.07
G2	22.70 ± 0.01	2.05 ± 0.32	1.64 ± 0.23	4.95 ± 0.09	158.72 ± 0.66	289.82 ± 1.06	1007.39 ± 0.52	287.68 ± 6.74	10.13 ± 0.09	2.62 ± 0.71	2.41 ± 0.31	5.51 ± 0.19	0.31 ± 0.02
G3	25.44 ± 0.43	1.15 ± 0.01	1.63 ± 0.34	6.66 ± 0.04	152.46 ± 3.33	297.86 ± 7.44	1022.57 ± 5.32	272.75 ± 3.97	8.84 ± 0.57	2.03 ± 0.17	1.94 ± 0.39	6.62 ± 0.31	0.33 ± 0.04
G4	21.34 ± 0.14	1.54 ± 0.16	1.41 ± 0.02	5.31 ± 0.01	150.29 ± 3.05	304.57 ± 0.32	1066.87 ± 6.66	260.48 ± 6.30	8.75 ± 0.21	2.15 ± 0.25	2.33 ± 0.20	6.70 ± 0.17	0.23 ± 0.06
G5	22.85 ± 0.01	2.26 ± 0.29	1.95 ± 0.06	4.48 ± 0.11	148.37 ± 3.63	280.77 ± 2.48	1026.49 ± 2.01	215.37 ± 0.90	8.81 ± 0.95	2.32 ± 0.43	2.18 ± 0.44	5.28 ± 0.15	0.42 ± 0.13
G6	21.63 ± 0.17	2.21 ± 0.07	1.97 ± 0.74	5.11 ± 0.05	158.53 ± 3.22	291.14 ± 6.06	1011.89 ± 7.43	280.73 ± 5.84	9.81 ± 0.14	2.43 ± 0.22	1.86 ± 0.14	5.70 ± 0.13	0.33 ± 0.01
G7	22.47 ± 0.40	1.15 ± 0.03	1.84 ± 0.55	6.20 ± 0.04	161.89 ± 2.11	288.31 ± 4.50	1028.28 ± 6.10	250.46 ± 5.47	8.84 ± 1.00	2.67 ± 0.28	2.35 ± 0.13	6.36 ± 0.28	0.22 ± 0.07
G8	23.37 ± 0.21	1.00 ± 0.01	2.82 ± 0.94	6.39 ± 0.01	165.47 ± 0.34	310.73 ± 6.95	1049.89 ± 2.19	213.49 ± 4.11	10.17 ± 0.16	2.24 ± 0.42	2.80 ± 0.11	6.80 ± 0.36	0.35 ± 0.06
G9	20.80 ± 0.19	1.17 ± 0.14	1.85 ± 0.93	4.24 ± 0.00	150.23 ± 2.35	296.00 ± 1.69	1000.38 ± 9.89	200.19 ± 2.81	8.97 ± 0.78	2.03 ± 0.11	1.76 ± 0.15	6.13 ± 0.13	0.25 ± 0.07
G10	21.24 ± 0.15	1.93 ± 0.02	2.36 ± 0.38	4.06 ± 0.00	170.25 ± 0.27	270.05 ± 6.04	1045.68 ± 5.99	203.66 ± 4.13	8.39 ± 0.87	2.39 ± 0.40	2.25 ± 0.16	6.86 ± 0.48	0.36 ± 0.03
G11	22.41 ± 0.09	1.23 ± 0.17	1.56 ± 0.35	5.30 ± 0.06	152.97 ± 1.51	275.95 ± 2.58	1041.15 ± 7.04	230.61 ± 4.08	10.09 ± 0.04	3.29 ± 0.99	2.60 ± 0.27	7.91 ± 0.46	0.26 ± 0.02
G12	22.71 ± 0.24	1.73 ± 0.03	2.04 ± 0.70	6.29 ± 0.10	143.64 ± 1.64	298.51 ± 7.30	1074.38 ± 9.51	261.19 ± 3.53	7.59 ± 0.30	2.52 ± 0.58	2.07 ± 0.28	7.34 ± 0.81	0.32 ± 0.15
G13	20.07 ± 0.41	2.12 ± 0.05	1.85 ± 0.74	4.66 ± 0.04	140.67 ± 0.20	300.78 ± 1.02	1019.38 ± 5.84	273.92 ± 1.40	8.90 ± 0.77	2.31 ± 0.33	2.10 ± 0.11	7.74 ± 0.74	0.34 ± 0.02
G14	21.73 ± 0.18	1.47 ± 0.01	1.02 ± 0.23	5.24 ± 0.07	149.79 ± 1.79	281.15 ± 6.73	1000.24 ± 4.16	254.69 ± 5.30	8.36 ± 0.53	2.69 ± 0.51	1.58 ± 0.22	7.78 ± 0.88	0.35 ± 0.10
G15	22.54 ± 0.29	1.65 ± 0.03	1.65 ± 0.22	6.24 ± 0.07	152.98 ± 3.58	293.65 ± 7.49	1005.21 ± 9.94	219.86 ± 0.46	9.14 ± 0.64	2.31 ± 0.28	1.85 ± 0.32	5.53 ± 0.31	0.27 ± 0.03
G16	22.78 ± 0.34	1.23 ± 0.15	1.28 ± 0.12	7.07 ± 0.03	142.52 ± 0.74	296.73 ± 0.62	1052.18 ± 6.02	241.47 ± 2.76	7.63 ± 0.34	1.99 ± 0.20	2.38 ± 0.15	5.83 ± 0.24	0.31 ± 0.03
G17	21.93 ± 0.13	0.98 ± 0.03	1.80 ± 0.11	4.30 ± 0.03	152.11 ± 2.45	273.55 ± 1.00	1011.41 ± 4.21	210.83 ± 4.17	9.66 ± 0.32	2.54 ± 0.43	2.17 ± 0.47	7.11 ± 0.12	0.32 ± 0.04
G18	22.62 ± 0.17	1.12 ± 0.23	1.64 ± 0.25	5.37 ± 0.16	155.84 ± 3.65	295.15 ± 1.08	1038.73 ± 3.78	294.54 ± 2.45	6.12 ± 0.38	2.42 ± 0.17	1.49 ± 0.28	8.95 ± 0.46	0.29 ± 0.04
G19	23.01 ± 0.13	2.36 ± 0.48	1.33 ± 0.21	4.05 ± 0.02	158.35 ± 3.21	298.76 ± 2.02	1002.31 ± 2.71	213.74 ± 3.89	8.88 ± 0.45	1.97 ± 0.26	1.69 ± 0.36	7.04 ± 0.32	0.35 ± 0.05
G20	21.53 ± 0.12	1.18 ± 0.39	1.60 ± 0.32	5.44 ± 0.04	168.12 ± 3.32	284.64 ± 0.59	1045.37 ± 9.25	233.18 ± 5.22	9.68 ± 0.27	1.86 ± 0.33	2.42 ± 0.30	6.69 ± 0.37	0.32 ± 0.04
G21	22.33 ± 0.19	1.17 ± 0.01	1.36 ± 0.37	6.51 ± 0.03	145.27 ± 3.63	296.11 ± 4.47	1031.74 ± 8.05	284.59 ± 1.33	6.70 ± 0.41	2.32 ± 0.34	2.63 ± 0.22	7.55 ± 0.55	0.22 ± 0.08
G22	24.98 ± 0.16	1.05 ± 0.08	2.15 ± 0.21	7.35 ± 0.05	169.72 ± 1.32	305.27 ± 4.77	1055.91 ± 1.10	298.66 ± 1.55	10.71 ± 0.29	2.26 ± 0.31	2.62 ± 0.36	6.28 ± 0.43	0.22 ± 0.06
G23	22.60 ± 0.25	1.87 ± 0.03	2.20 ± 0.21	5.94 ± 0.06	140.52 ± 2.78	297.04 ± 4.95	1000.35 ± 1.04	276.89 ± 5.33	6.63 ± 0.33	2.84 ± 0.61	3.10 ± 0.29	8.46 ± 0.55	0.28 ± 0.01
G24	21.42 ± 0.40	1.19 ± 0.10	1.86 ± 0.74	6.24 ± 0.02	153.76 ± 0.24	285.45 ± 4.75	1010.82 ± 8.94	300.96 ± 2.66	8.26 ± 0.60	2.31 ± 0.20	1.98 ± 0.41	6.37 ± 0.35	0.30 ± 0.01
G25	23.71 ± 0.04	1.29 ± 0.01	1.98 ± 0.21	4.79 ± 0.05	140.94 ± 2.05	276.99 ± 3.36	1001.78 ± 9.38	205.94 ± 4.49	7.43 ± 0.44	1.45 ± 0.32	2.10 ± 0.29	5.08 ± 0.56	0.29 ± 0.03
G26	21.48 ± 0.26	1.24 ± 0.02	1.58 ± 0.18	5.69 ± 0.00	150.83 ± 1.10	296.42 ± 4.17	1035.49 ± 2.16	237.19 ± 5.06	8.72 ± 0.56	2.31 ± 0.73	2.10 ± 0.26	7.49 ± 0.52	0.35 ± 0.03
G27	23.70 ± 0.05	1.20 ± 0.11	2.57 ± 0.28	5.53 ± 0.05	153.22 ± 2.07	304.71 ± 4.44	1057.46 ± 1.10	245.94 ± 5.25	9.75 ± 0.75	1.56 ± 0.53	1.96 ± 0.57	6.88 ± 0.35	0.27 ± 0.08
G28	22.60 ± 0.08	1.38 ± 0.07	1.82 ± 0.10	5.69 ± 0.06	146.53 ± 1.22	272.15 ± 1.42	1003.86 ± 5.75	258.33 ± 4.97	7.56 ± 0.38	2.52 ± 0.16	2.39 ± 0.31	8.72 ± 0.55	0.45 ± 0.21
G29	22.81 ± 0.20	1.14 ± 1.45	2.13 ± 0.29	6.37 ± 0.01	153.00 ± 0.96	303.49 ± 1.74	1015.43 ± 3.17	281.19 ± 1.61	6.40 ± 0.49	2.04 ± 0.18	2.51 ± 0.51	7.93 ± 0.27	0.19 ± 0.08
G30	21.80 ± 0.17	0.97 ± 0.10	1.84 ± 0.16	5.62 ± 0.06	168.14 ± 2.89	309.89 ± 1.61	1085.96 ± 1.13	262.19 ± 2.87	7.20 ± 0.31	2.79 ± 0.12	2.03 ± 0.41	7.59 ± 0.24	0.32 ± 0.02
Mean	22.48	1.50	1.82	5.57	154.10	292.81	1030.07	252.03	8.59	2.33	2.20	6.84	0.30
Lowest	20.07	0.98	1.02	4.05	140.52	270.05	1000.24	200.19	6.12	1.45	1.49	4.83	0.19
Highest	25.44	2.36	2.82	7.35	170.25	310.73	1085.96	300.96	10.71	3.29	3.10	8.95	0.45
SD	1.13	0.43	0.38	0.87	8.87	11.44	24.15	31.11	1.20	0.37	0.36	1.05	0.06
CV (%)	5.01	29.98	20.72	15.54	5.76	3.91	2.34	12.34	13.99	15.82	16.25	15.39	19.21

Mean values of three environments viz., E1, E2, and E3 with SE (Standard Error) B Boron, Mg Magnesium, P Phosphorus, K Potassium, Ca Calcium, Mn Manganese, Fe Iron, Cu Copper, Zn Zinc, Mo Molybdenum.

A wide range of variation is observed for B (4.05–7.35 mg/100 g), Mg (140.52–170.25 mg/100 g), P (270.05–310.73 mg/100 g), K (1000.24–1085.96 mg/100 g), Ca (200.19–300.96 mg/100 g), Mn (6.12–10.71 mg/100 g), Fe (1.45–3.29 mg/100 g), Cu (1.49–3.10 mg/100 g), Zn (4.83–8.95 mg/100 g), and Mo (0.19–0.45 mg/100 g). The genotype G22 possessed the highest B (7.35 mg/100 g) and Mn (10.71 mg/100 g). In G22, the contents of B and Mn are stable across environments indicating its potential as a donor. Likewise, the genotypes showed superior performance for Mg, P, K, Ca, Fe, Cu, Zn, and Mo are G10 (170.25 mg/100 g), G8 (310.73 mg/100 g), G30 (1085.96 mg/100 g), G24 (300.96 mg/100 g), G11 (3.29 mg/100 g), G23 (3.10 mg/100 g), G18 (8.95 mg/100 g), and G28 (0.45 mg/100 g) respectively. This comprehensive study on nutrient profiling in horse gram helped to identify elite genotypes consistently exhibiting higher nutrient content across diverse locations. These promising genotypes are invaluable resources, strategically empowering horse gram breeding programs for nutrient enhancement.

#### Understanding the relationship between the yield and quality traits

In a classical yield and quality improvement plant breeding program, it is generally believed or proven that a simultaneous enhancement of yield and quality is seldom accomplished due to negative associations and other related factors. Raatz^13^ reported a negative relationship between yield and quality. The genetic materials of the current experiment are derived from the variety PAIYUR 2 through an induced mutagenesis program. The practical utility of these mutants with a yield compromise over the parent is limited. Therefore, to ascertain the influence of improved yield with quality traits in the identified good yielding and stable mutants (GYSM), and vice versa in the macronutrient, and micronutrient specific mutants (MaNSM/MiNSM), the following analyses were made. In comparison to the parent PAIYUR 2, (i) the comparative macro and micronutrient supremacy of MaNSM and MiNSM; (ii) the comparative seed yielding supremacy of MaNSM and MiNSM; (iii) comparative seed yielding potential and macro and micronutrient contents of identified GYSM; and (iv) combined and comparative macro and micronutrient supremacy of MaNSM and MiNSM over the parent PAIYUR 2. The purposes of these analyses are, (i) to quantify the percentage increase of macro and micronutrients of MaNSM and MiNSM over the parent, (ii) to understand the relationship between increased nutrient contents with seed yield in MaNSM and MiNSM, (iii) to comprehend the supremacy of GYSM for yield and nutrient contents over the parent, and (iv) to estimate the comparative (with PAIYUR 2) influence of one enhanced macro/micro nutrient with other in MaNSM and MiNSM.

##### Analysis (i)

Comparison of nutrient levels between parent and MaNSM and MiNSM.

In MaNSM, all three genetic stocks had an advantage for macronutrients over the parent. The enhancements for protein, fat, and fibre are 16.70% (G3), 43.90% (G19), and 43.88% (G8) respectively (Supplementary Fig. S7a). A pronounced advantage was noticed for all the nutrients except Mg and P in MiNSM. The increased percentages are 30.83, 48.71, 14.79, 17.70, 52.49, 17.85, and 41.67 over the parent for B, Mn, Ca, Fe, Cu, Zn, and Mo respectively. A minimum percentage of enhancement was noticed for P (0.27) while the maximum was noticed for Cu (52.49) (Supplementary Fig. S7b). Earlier, Raina et al.^29^ reported enhanced nutrient content in the mutant lines of cowpeas. Therefore, MaNSM/MiNSM justifies their purpose of identification and can further be utilized in the targeted horse gram improvement program.

##### Analysis (ii)

Relationship between increased nutrient contents and seed yield in MaNSM and MiNSM.

The seed-yielding potentials of MaNSM and MiNSM were compared with the parent PAIYUR 2 (Supplementary Fig. S8) to understand the influence of increased nutrient contents on yield. In MaNSM, enhanced protein and fibre contents do not influence the seed-yielding potential while a yield compromise with enhanced fat (− 9.50%) is noticed. A positive relationship between yield increase with protein and fibre contents is established (Supplementary Fig. S8a). In MiNSM, yield compromise for most of the nutrients is observed except B (G22: + 32.49%), Mn (G22: + 32.49%), and P (G8: + 29.59%). The yield compromise levels for Mg, Ca, Fe, Cu, Zn, and Mo are − 2.64%, − 16.65%, − 1.65%, − 20.99%, − 9.76%, and − 16.78% respectively (Supplementary Fig. S8b). Further investigations are required to decipher the relationship between enhanced protein, fibre, B, Mn, and P with the improved yield to identify/fix relevant markers.

##### Analysis (iii)

Supremacy of GYSM for yield and nutrient contents over the parent.

This analysis helps to understand the supremacy of GYSM for yield and nutrient contents over the parent (Supplementary Fig. S9). The GYSM possessed a yield advantage of 42.99% (G1), 34.63% (G25), 28.68% (G3), and 30.59% (G27) than PAIYUR 2 (Supplementary Fig. S9a). For the macronutrients, a positive influence of increased yield on protein content (r^2^ = 0.69) is witnessed (Supplementary Fig. S10a) while a negative relationship between yield and fibre (r^2^ = − 0.16) (except G27) and fat (except G1) is established. The values Earlier, Csajbók et al.^30^ and Jarecki and Migut^12^ explained that protein yield in legumes depends on seed yield rather than the per se protein content. For the micronutrients, on a majority, a negative relationship with increased yield is observed except for Mn (r^2^ = 0.63), Cu (r^2^ = 0.46), and B (r^2^ = 0.01) (Supplementary Fig. S10b). The combined results of GYSM with nutrients indicate that the genotypes G1 and G25 possess a positivity between yield and nutrients and therefore can further be utilized for commercial exploitation.

##### Analysis (iv)

Interrelationship between nutrients in MaNSM and MiNSM: A comparison with parent.

In this analysis, the inter-relationship between PAIYUR 2 and MaNSM and MiNSM for nutrients was established (Supplementary Fig. S11). In MaNSM, it is observed that increased protein content (+ 16.70%) in G3 compromises the fat (− 29.88%) and fibre (− 16.84%) contents. The enhanced fat (+ 43.90%) in G19 made a compromise for the fibre (− 32.14%) but not the protein (+ 5.55%) and increased fibre in G8 (+ 43.88%) reduced the fat (− 39.02%) and showed a slight increase in protein (+ 7.20%) (Supplementary Fig. S11a). Jarecki and Migut^12^ reported a significant and negative link between protein and fat content. Upon analyzing the micronutrient perspective, it is inferred that the majority of the stocks expressed a negative relationship with other nutrients except for Mn, Cu, and B (Supplementary Fig. S11b). Increased Cu is positively associated with B, Mn, Mg, P, Fe, and Mo. Enhanced Mn content is positively associated with Mg, P, Ca, Fe, and Mo, likewise, B is with Mn, P, Ca, Cu, and Mo. Therefore, in MiNSM, the genotypes G22 (B and Mn) and G23 (Cu) can further be considered for exploitation.

#### Physical and cooking properties

Ascertaining seed physical properties like density, size dimensions, and shape help to fabricate various machines employed in sowing, harvesting, post-harvest processing, and value addition^14^. Therefore, the seed dimension factors length, breadth, and their ratio, bulk density, 1000 grain weight, and specific gravity were ascertained for the identified mutants (Supplementary Table. 2). No significant variation was observed between the mutants and PAIYUR 2 for the seed length, however, G25 had a higher breadth (0.37 ± 0.006) which eventually implicated in the L/B ratio (1.47 ± 0.024). In case of further promotion of G25 for commercial cultivation, variety-specific machine designing is warranted, while for G1 the machines already being utilized for the variety PAIYUR 2 can be utilized.

The SG of seeds is directly related to germinability. The higher the SG, the higher the germination percentage^31^. The SG of G1 (1.61 ± 0.009) and G25 (1.59 ± 0.002) are higher than PAIYUR 2 (1.43 ± 0.004). The germination capacity of G1 and G25 are higher than PAIYUR 2 both under laboratory and field conditions. Seed size determines the BD^32^. BD influences the storability of grains and thus affects the quality of stored grains in the food chain^33^. The BD of G1 (0.91 ± 0.011) is higher than the other tested genotypes which has an economic advantage. Flours with high BD increase fat absorption rate which is a desirable flour trait for pastry and baked product preparation while G25 can be considered for preparation of weaning foods^34^. The mutants also possessed higher TGW that ultimately had a positive influence on the seed-yielding potential (Supplementary Table. 2).

In the human diet, food legumes are consumed along with cereals to cater to carbohydrate, protein, and mineral requirements. Food legumes are cooked before serving to improve their nutritional quality and palatability. Over/insufficient legume cooking affects the nutrient quality and intake availability. In legumes, during cooking, the time taken by the cotyledon cells to separate satisfactorily is referred to as cooking time (CT). Studies on legume cookability revealed relationships between seed physio-chemical properties and CT. Horse gram seeds have strongly adhered cotyledons^35^ that increase the CT. Dehusking and splitting of cotyledons are practiced to reduce the CT, however, in horse gram, these practices are not effective. In horse gram, lesser information on cooking pre-soaking treatments to reduce CT is available and further very rarely practiced due to the cost of horse gram grain in the market (the per kg cost of horse gram grain in the Indian market is one-third of the cost of red gram or mung/urd beans). Therefore, the identification of horse gram with good yield and cookability traits is important.

The WA influences the CT. The WA is directly influenced by the seed coat properties. Usually, legumes are characterized by hard seed coats; horse gram is not an exemption. In this study, the CT of tested genotypes G1 and G25 had the CT of 100 and 101 min respectively, which is lesser than the parent (104 min) (Supplementary Table. 3). A negative relationship between the WA and CT is established. In G1 and G25 the WA were 18.5 and 16 ml respectively while it was 15 ml for the parent. The correlation r^2^ value between CT and WA is -0.53 (Supplementary Fig. S12). However, earlier, a weaker linkage^36,37^, and a negative association^38^ were reported. Also, a negative correlation between CT and cooked weight (r^2^ = − 0.88) is noticed. A positive correlation between CT and LER (r^2^ = 0.99), BER (r^2^ = 0.76), and TSS (r^2^ = 0.91) was established. Enhanced LER and BER might have contributed to a higher cooked weight in G1 (20.590 ± 0.350 g) and G25 (20.125 ± 0.545 g) respectively than the parent.

#### Amino acid fractionation

The AA profiling through LC–MS–MS was done for the two identified mutants G1 and G25 and the results were compared to the parent PAIUYR 2 to understand the impact of increased yield and protein on essential (EAA) and non-essential AA (NEAA) contents and to assess the protein value of the identified stable mutants. The impacts were analyzed in four ways (i) changes in the EAA in comparison to total AA, (ii) changes in the EAA in comparison to total EAA, (iii) changes in the NEAA in comparison to total AA, and (iv) changes in the NEAA in comparison to total NEAA. It is observed that induced mutagenesis decreased the NEAA content in the mutants, however, a genotype-dependent effect increment/decrement effect was noticed. In the EAA, lysine serves as a major protein building block. It ensures proper human growth through carnitine production. A proper fatty acid conversion into energy molecules depends on carnitine synthesis. This conversion maintains cholesterol at its low level thereby avoiding coronary health issues and reducing obesity. Lysine strengthens the bone framework in the human by improving calcium absorption and collagen formation. Regular and sufficient calcium intake reduces the risk of diabetes (type II). Collagen is also an important component in the maintenance of the skin, tendons, and cartilage. Dietary lysine and calcium intake satisfy human requirements. This study revealed a dual advantage of good lysine (Supplementary Fig. S13) and calcium content (Table 4) in the mutant G25 which offers the scope of functional food preparations. The mutant G25 had higher lysine content than the parent and G1. Its lysine content accounts for 10.19% of the total AA content (Supplementary Fig. S13) and 44.58% of the EAA (Supplementary Fig. S14). The lysine significance in horse gram is well reported earlier^39^. The order of other EAA are phenylalanine, threonine, valine, leucine, and histidine. The percentages to the total AA for G25 are 7.73%, 1.35%, 1.31%, 0.87%, and 0.71%; for G1 the contents are 7.78%, 1.32%, 1.30%, 0.81%, and 0.65%; and for the parent they are 8.05%, 1.38%, 1.29%, 0.89%, and 0.64% respectively. Legumes are generally deficient in tryptophan and methionine. The tested horse gram genotypes in this experiment also lacked tryptophan and isoleucine however interestingly methionine was detected which is nutraceutically considered significant. It constitutes 0.65 to 0.71% of total AA and 3.01 to 3.12% of total EAA (Supplementary Fig. S13,S14). The cascade of NEAA is constituted mainly by glutamic acid, and aspartic acid, and followed by glutamine, arginine, glycine, proline, serine, alanine, tyrosine, and cystine (Supplementary Fig. S15,S16).

## Conclusion

These multiple-environment-based investigations helped to (i) identify a few high-yielding and stable horse gram genotypes (G1, G3, G25, and G27) and (ii) nutrient-specific genetic stocks. These high-yielding and stable genotypes can be utilized for variety release after on-farm trials. The nutrient-specific genetic stocks shall be considered as donors in the quality improvement programs.

## Materials and methods

### Plant genetic materials

The genetically pure seeds of the two well-known horse gram varieties PAIYUR 2 and CRIDA 1–18 R were obtained from the Regional Research Station, Paiyur, Tamil Nadu, India, and Central Research Institute for Dryland Agriculture, Hyderabad, India respectively, and mutated in 32 mutagenic combinations comprising physical mutagens (electron beam and gamma rays) and a chemical mutagen (EMS). The source of gamma rays is Cobalt-60 (^60^Co) and the concentrations include gamma rays (100–400 Gy), electron beam (100–400 Gy), 0.3% EMS, and their combinations. The seeds were irradiated at the mutation facility of the Board of Research in Nuclear Sciences** (**BRNS), Government of India (GoI), India. The early mutagenic generations were carefully evaluated from the cropping years 2016 to 2019 using standard plant breeding procedures^5^*.* Currently, with the support of a Department of Science and Technology-Science and Engineering Research Board- GoI-funded project, the useful homozygous lines are being evaluated in various plant breeding trials. During the cropping year 2020, the level of variability evolved through the induced mutagenesis in these populations for yield-related traits was documented by Pushpayazhini et al.^6^ and based on her findings, 29 promising mutants were selected for further utilization. The genetically pure seeds of these 29 mutants were multiplied sufficiently during the cropping season 2021. To understand the commercial breeding potency of these earmarked 29 mutants (G1 to G29) (Supplementary Table. 1) along with the check PAIYUR 2 (G30) therefore were utilized in the stability and nutritional profiling experiments.

### Location, design, and data documentation

Horse gram is a short-day, and photosensitive crop, and therefore the stability of the genotypes was assessed over three different environments during the *Rabi* season, in the cropping year 2022. The environments are the Department of Pulses, Centre for Plant Breeding and Genetics, Tamil Nadu Agricultural University, Coimbatore, Tamil Nadu (11.02 °N and 76.92 °E): E1, Sugarcane Research Station, Tamil Nadu Agricultural University, Melalathur, Vellore (12.91°N and 78.87 °E): E2, and a farmer’s experimental field located at Krishnagiri district, Tamil Nadu (12.34 °N and 78.13 °E): E3. The E3 was laid out to assess the real-time performance of the genotypes at the farmer’s practice. The performances of the genotypes were assessed on a Randomized Block Design (RBD) with three replications. All the required agronomic and crop protection practices were adopted to ensure healthy crop growth. Seeds were sown in 5-m rows with a spacing of 45 × 15 cm. The number of rows per genotype per replication was six. Ten randomly selected plants from each replication were utilized for documenting various yield-contributing traits at appropriate growth stages. The considered traits are days to maturity (DMY), number of clusters per plant (NC), number of pods per plant (NP), number of seeds per pod (NS), and yield per hectare (YPH). DMY was noted at the relevant growth stage, while other characteristics were documented during and after harvest.

### Statistical analyses for stability estimation

Stability analyses were performed using AMMI and GGE models in R studio (METAN package—version 4.1.1.)^40^. Initially, ANOVA (analyses of variance) for AMMI was performed to analyze the variability of the genetic material. The principal components with maximum variation were used to plot AMMI biplots. These biplots along with GGE plots were used to visualize the GEI. AMMI-based statistical parameter like AMMI Stability value (ASV) is calculated using the formula given by Purchase et al.^41^. The Genotype Selection Index (GSI) is worked based on Farshadfar et al.^20^. Ranking and evaluation of genotypes are carried out using a mean *vs*. stability biplot. Test environments are compared with the help of the discriminativeness *vs*. representativeness biplots. Stable and specific genotype recommendation is done using the which-won-where biplot.

### Assessment of macro and micronutrients

In an environment, replication-wise, sufficient quantities of seeds from genotypes after drying to adequate moisture content were collected. For a genotype, the seeds of three replications were pooled. For nutrient profiling, samples were drawn from the pooled seeds. Macronutrients like crude protein, and crude fiber, were estimated by adopting the procedures advocated by Lynch and Lynch^42^, and Maynard^43^. Crude fat was estimated with the help of the Soxhlet apparatus. Micronutrients magnesium, potassium, manganese, zinc, boron, phosphorus, calcium, iron, copper, and molybdenum were estimated using Inductively Coupled Plasma-Mass-Spectrometry (Model: Thermo Scientific™ iCAP™ RQ; Type: Single Quadrupole ICP-MS; Dynamic Range: > 9 orders of magnitude (< 1—> 1·109 cps); Hertz: 2 MHz). The data sets were analyzed using Microsoft Excel 2016 (Version 2306) and SPSS software (version 28.0.1).

### Estimation of the relationship between yield and nutrients and inter se nutrients

Pearson’s correlation coefficient (R studio) and percentage comparison analyses (Microsoft Excel 2016 (Version 2306) were utilized. The correlograms with the significance values were constructed using R studio.

### Estimation of physical and cooking properties and amino acid profiling

The mutants selected after stability, nutrient, and relationship analyses were utilized for these estimations to fix the seed and food-chain requirements.

### Estimation of physical properties

The seeds of parent PAIYUR 2 and identified mutants were cleaned manually to remove the extraneous material. The physical parameters grain length (L) and breadth (B) (graphical method), length/breadth ratio (L/B ratio)^44^, 1000 grain weight (TGW)^45^, bulk density (BD)^46^, specific gravity (SG) (weight/volume), and germination percentage^47^ were assessed in five replications. The averaged values were utilized for analyses.

### Estimation of cooking properties

The seeds were washed sufficiently with running tap water to remove seed coat-adhering residues. The cooking qualities length elongation ratio (LER), breadth elongation ratio (BER), cooking time (CT)^48,49^, total soluble salts (T.S.S) (using a hand-held refractometer), and water absorption ratio (WA) were assessed. These experiments were replicated twice. The averaged values were utilized for analyses.

### Statistical analysis for physical and cooking properties

The data sets were analyzed using R studio (Pearson’s correlation coefficient), and Microsoft Excel 2016 (Version 2306) & SPSS software (version 28.0.1) (first-order statistics). The correlograms with the significance values were constructed using R studio.

### Amino acid profiling

#### Sample preparation

Grains of horse gram genotypes (PAIYUR 2, G1, and G25) were ground and passed through a sieve (No:60 as per Bureau of Indian Standards). The fine flours were packed in air-tight containers and stored in the refrigerator.

#### Sample extraction

Approximately 500 mg of homogenized samples were weighed in 20 mL amber-colored bottles. An aliquot of 2.50 mL of 6N aqueous HCl and 4% phenol were added to the bottles and kept at 110 °C for 6 h. After cooling, the samples were transferred to 50 ml of standard flasks and the volume was made up using 0.1N HCL and centrifuged at 10,000 rpm for 10 min at 4 °C, the supernatants were filtered through 0.22 μm filter paper and injected onto LC–MS/MS. Need-based dilution was done with 0.1N HCL.

#### LC–MS/MS analysis

The analysis was carried out on a Waters Acquity TQD system with a triple quadrupole mass spectrometer equipped with an electrospray ionization (ESI +) probe. A 40 µL aliquot was injected through an auto-sampler. Separation was achieved using a reverse phase Phenomenex kinetex C18 100°A, 2.6 µM, 4.6 × 100 mm column; the flow rate: 0.350 mL/min and stop time: 7 min; and the column thermostats were 40 °C). The mobile phase A was composed of 30 mM Ammonium acetate in water at pH 6.00 with acetic acid and mobile Phase B was composed of 0.1% acetic acid in acetonitrile. The solvent program was gradient (The percentage of A and B (v/v) were 0–1.8 min: 98 and 2%; 1.9–2.4 min: 60 and 40%; 2.5–2.9 min: 10 and 90%; 3.0–4.0 min: 10 and 90%; 4.1–7.0 min: 98 and 2%). The MS/MS conditions were capillary: 2.00 kV; cone:15 V; extractor:2 V; RF Lens:0.3 V; source temperature: 150 °C; desolvation temperature: 400 °C; gas flow 900 (L/hr); and cone flow 30 (L/hr). Estimation was performed using selective ion monitoring (SIM) with a dwell of 0.025 s. The precision of the instrument is monitored by checking the deviation of the concentration of a calibration check solution (25 µg of AA/mL) or LOQ/MDL. It is performed after the calibration of every 10 samples. Calibration curves were constructed by analysis of standard solutions prepared both in solvent and matrix solution at four (0.5–50 µM) and three (5–50 µM) concentrations, respectively. Instrumental detection limits (IDLs) corresponded to a concentration with a signal-to-noise ratio of 3. Method accuracy and precision were evaluated by analyzing unspiked and spiked horse gram samples at concentrations equivalent to the natural ones. The correlation coefficient (r^2^) was 0.9900. The amino acid content was estimated using the software Mass LYNXV4.1 supplied along with the instrument. The data were analyzed using Microsoft Excel 2016 (Version 2306). The results are represented as AA present in µg/g of horse gram seeds ± SE.

### Plant experiments

All the experiments in this manuscript were conducted in an indigenous and cultivated horse gram species (*Macrotyloma uniflorum*) by following relevant institutional, and national guidelines and legislation.

### Supplementary Information

Supplementary Information.

## Data Availability

All data documented in these experiments are published in this article.
