# Supplementary material for: Assessing the yield and nutrient potential of horse gram mutants (Macrotyloma uniflorum Lam. Verdc.) an underutilized legume through a multi-environment-based experiment
Source: Sci Rep. 2024 Jul 15;14:16305. doi: 10.1038/s41598-024-67282-5 (PMC11250823; doi:10.1038/s41598-024-67282-5)
Supplement: Supplementary file 1 — Supplementary Information. [file 41598_2024_67282_MOESM1_ESM.pdf]

Assessing the yield and nutrient potential of horse gram mutants (*Macrotyloma uniflorum* Lam. Verdc.) an underutilized legume through a multi-environment-based experiment

Sumaiya Sulthana Jafarullakhan[1], Vaishnavi Vijayakumar[1], Kundan Veer Singh[1], Naaganoor Ananthan Saravanan[2], Veeranan Arun Giridhari[3], Sivakumar Rathinavelu[4], Balaji Kannan[5], Vanniarajan Chockalingam [6], Raveendran Muthurajan[7], Karthikeyan Subburamu[3], Selvaraju Kanagarajan[8*] & Sudhagar Rajaprakasam[1*]

Supplementary Tables

Supplementary Table 1. Details of experimented mutants and check PAIYUR 2

S.No	Genotypes	Abbreviation	Mutagen dose*
**1**	TNAU-HG-007	G1	200 Gy (GR)
**2**	TNAU-HG-031	G2	100 Gy (EB)
**3**	TNAU-HG-070	G3	100 Gy (GR)
**4**	TNAU-HG-034	G4	100 Gy (GR)
**5**	TNAU-HG-019	G5	300 Gy (GR)
**6**	TNAU-HG-027	G6	100 Gy (EB)
**7**	TNAU-HG-049	G7	200 Gy (GR) + 0.3% EMS
**8**	TNAU-HG-018	G8	100 Gy (GR)
**9**	TNAU-HG-062	G9	100 Gy (EB) + 0.3% EMS
**10**	TNAU-HG-057	G10	300 Gy (GR)
**11**	TNAU-HG-073	G11	100 Gy (EB) + 100 Gy (GR)
**12**	TNAU-HG-083	G12	200 Gy (GR)
**13**	TNAU-HG-036	G13	100 Gy (GR) + 0.3% EMS
**14**	TNAU-HG-030	G14	100 Gy (GR)
**15**	TNAU-HG-082	G15	300 Gy (GR)
**16**	TNAU-HG-025	G16	100 Gy (EB)
**17**	TNAU-HG-053	G17	200 Gy (GR) + 100 Gy (EB)
**18**	TNAU-HG-016	G18	300 Gy (GR)
**19**	TNAU-HG-071	G19	100 Gy (EB)
**20**	TNAU-HG-075	G20	200 Gy (GR) + 0.3% EMS
**21**	TNAU-HG-065	G21	300 Gy (GR)
**22**	TNAU-HG-089	G22	100 Gy (GR)
**23**	TNAU-HG-032	G23	100 Gy (EB) + 0.3% EMS
**24**	TNAU-HG-003	G24	200 Gy (GR)
**25**	TNAU-HG-081	G25	300 Gy (GR)
**26**	TNAU-HG-011	G26	100 Gy (EB)
**27**	TNAU-HG-039	G27	200 Gy (GR)
**28**	TNAU-HG-076	G28	100 Gy (GR)
**29**	TNAU-HG-044	G29	100 Gy (GR) + 0.3% EMS
**30**	PAIYUR 2	G30	Parent

*Mutagen dose from which the mutant evolved
Gy -Gray, GR -Gamma Ray, EB -Electron Beam, EMS -Ethyl Methane Sulphonate

Supplementary Table 2. Physical parameters for the identified stable genotypes *viz.*, G1, G25 & PAIYUR 2

Genotype	**L(cm)**	**B(cm)**	**L/B ratio**	**TGW (gm)**	**SG (wt./vol.)**	**Germination percent (%)**		**BD (g/ml)**
						Lab	Field	
G1	0.55±0.004	0.39±0.005	1.41±0.026	33.39±0.309	1.61±0.009	90.5±0.500	81.00±0.50	0.91±0.011
G25	0.55±0.004	0.37±0.006	1.47±0.024	33.26±0.468	1.59±0.002	90.0±0.000	80.50±0.50	0.86±0.002
PAIYUR 2	0.55±0.006	0.39±0.002	1.40±0.011	30.82±0.411	1.43±0.004	88.5±0.500	79.50±0.50	0.86±0.002
Mean	0.55	0.38	1.43	32.49	1.55	89.67	80.33	0.88
SD	0.01	0.01	0.04	1.45	0.10	1.04	0.76	0.03
CV(%)	1.82	2.46	2.80	4.45	6.61	1.16	0.95	2.96

L- Lenth, B-Breadth, L/B ratio-Length Breadth ratio, TGW-Thousand grain weight, SG- Specific gravity, BD- Bulk density

Supplementary Table 3. Cooking quality parameters for the identified stable genotypes *viz.*, G1, G25 & PAIYUR 2

**Treatment-Tap water**										
	L(cm) uncooked	L(cm) cooked	B(cm) uncooked	B (cm) cooked	T.S.S (Brix %)	LER (cm)	BER (cm)	CT (min.)	WA (ml)	CW (gm)
G1	0.550±0.010	0.755±0.005	0.380±0.010	0.520±0.010	10.250±0.050	1.365±0.035	1.360±0.010	100.000±0.000	18.500±0.500	20.590±0.350
G25	0.555±0.005	0.725±0.005	0.370±0.010	0.500±0.010	7.350±0.450	1.300±0.020	1.34±0.065	101.000±0.000	16.000±2.000	20.125±0.545
PAIYUR 2	0.550±0.010	0.695±0.005	0.390±0.000	0.475±0.005	6.650±0.550	1.260±0.030	1.215±0.015	104.000±0.000	15.000±1.000	18.825±0.725
Mean	0.552	0.725	0.380	0.498	8.083	1.308	1.307	101.667	16.500	19.847
SD	0.003	0.030	0.010	0.023	1.909	0.053	0.080	2.082	1.803	0.915
CV(%)	0.523	4.138	2.632	4.524	23.613	4.050	6.102	2.048	10.926	4.609

L-Length (cm), B-Breadth (cm), T.S.S -Total soluble sugars (Brix %), LER- Length elongation ratio (cm), BER- Breadth elongation ratio (cm), CT- Cooking time (min.), WA- Water Absorption (ml), CW- Cooked Weight (gm)

Assessing the yield and nutrient potential of horse gram mutants (*Macrotyloma uniflorum* Lam. Verdc.) an underutilized legume through a multi-environment-based experiment

Sumaiya Sulthana Jafarullakhan[1], Vaishnavi Vijayakumar[1], Kundan Veer Singh[1], Naaganoor Ananthan Saravanan[2], Veeranan Arun Giridhari[3], Sivakumar Rathinavelu[4], Balaji Kannan[5], Vanniarajan Chockalingam [6], Raveendran Muthurajan[7], Karthikeyan Subburamu[3], Selvaraju Kanagarajan[8*] & Sudhagar Rajaprakasam[1*]

Supplementary Figures

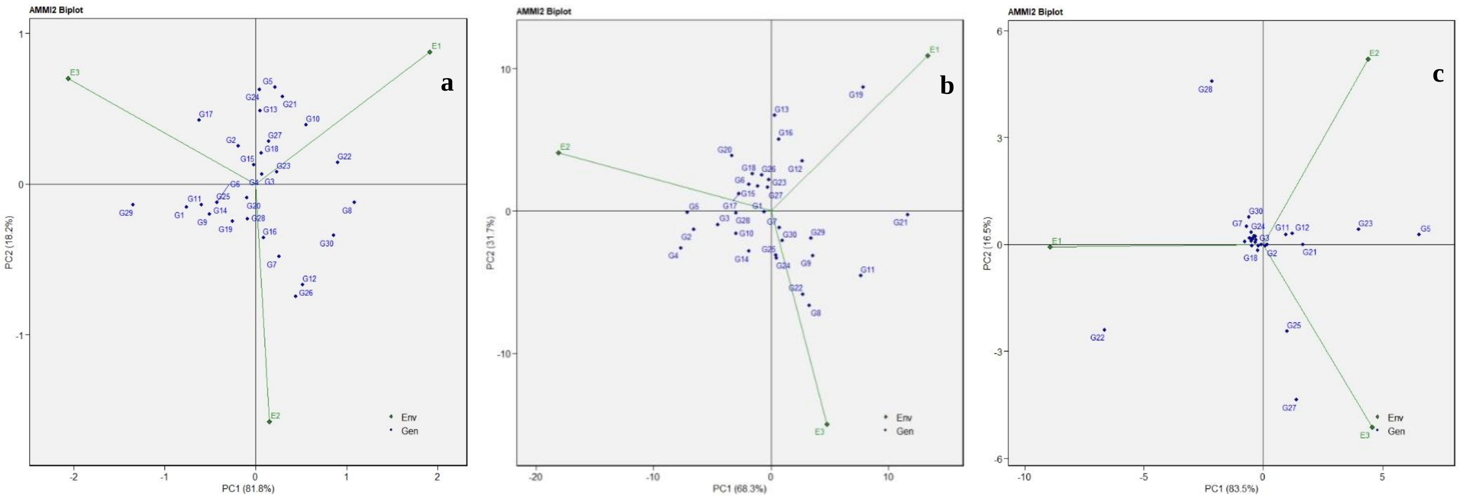

Supplementary Figure S1. The AMMI 2 biplot shows the first two Principal Component (PC) effects of both genotypes and GXE interaction of 30 horse gram genotypes in three different locations for the traits days to maturity-DMY (a), yield per hectare- YPH (b), and number of clusters per plant-NC (c)

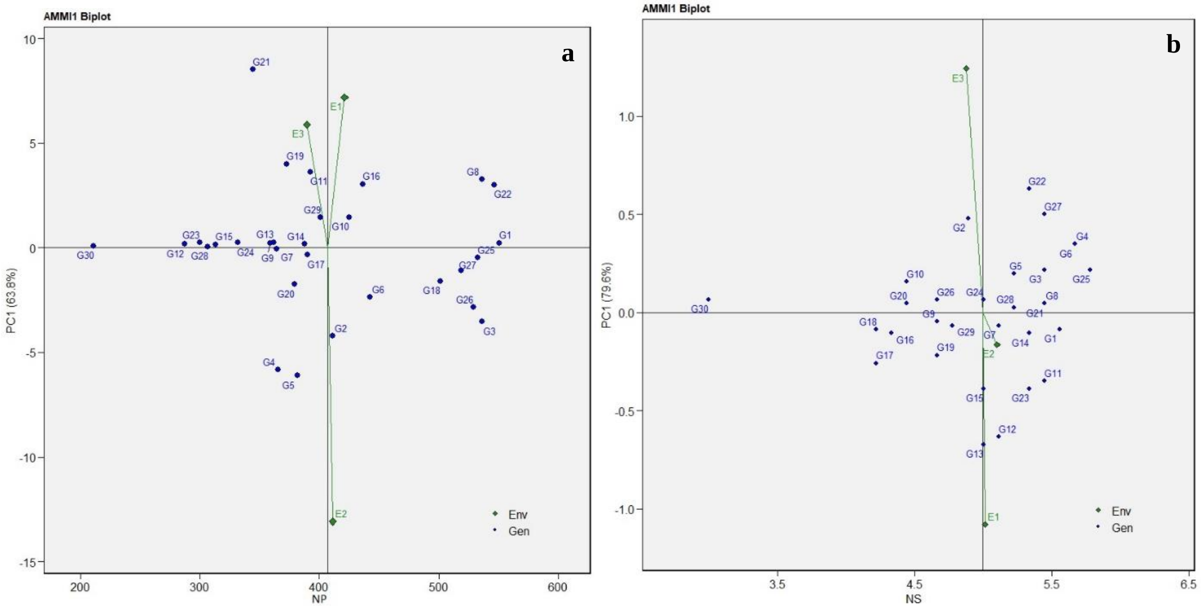

Supplementary Figure S2. The AMMI 1 biplot shows the first two Principal Component (PC) effects of both genotypes and GXE interaction of 30 horse gram genotypes in three different locations for the traits number of pods per plant-NP (a), number of seeds per pod-NS (b).

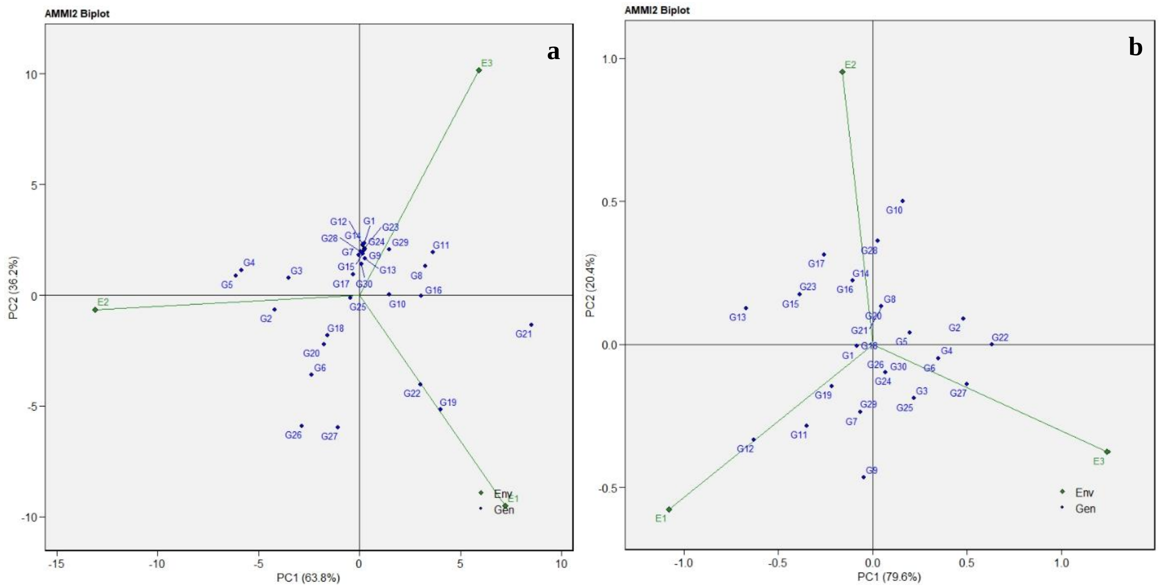

Supplementary Figure S3. The AMMI 2 biplot shows the main effect and Principal Component 1 (PC) of both genotypes and environments of 30 horse gram genotypes in three different locations for the traits number of pods per plant-NP (a), number of seeds per pod-NS (b).

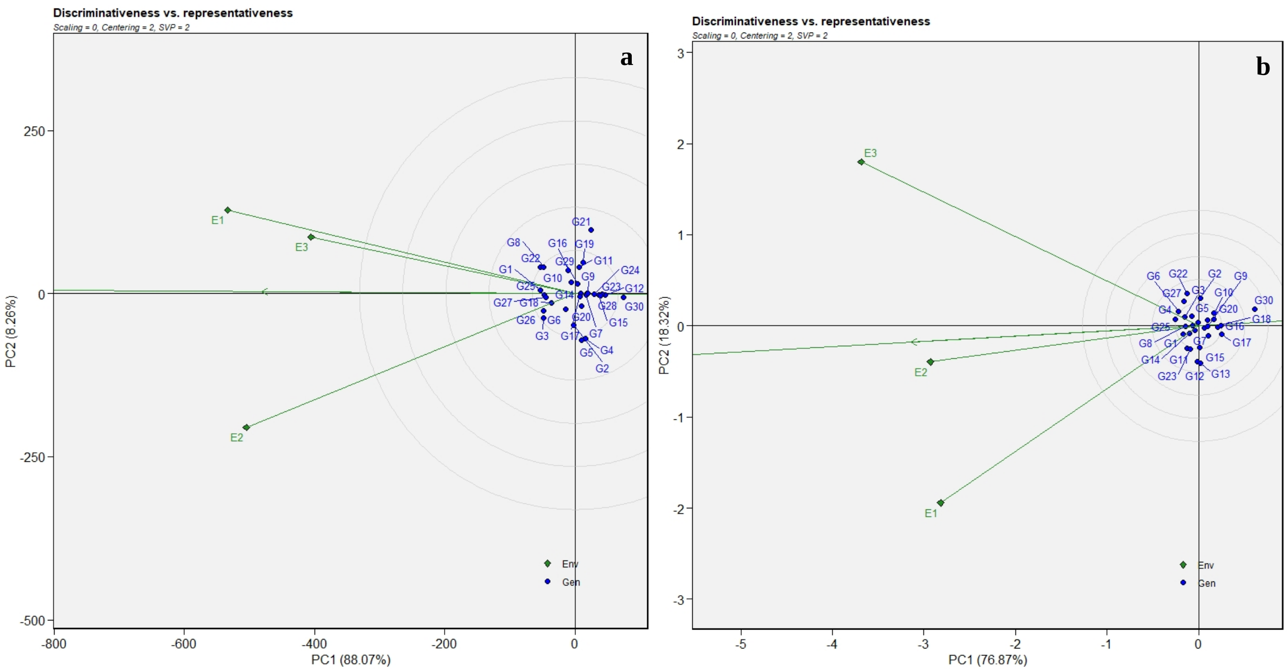

Supplementary Figure S4. Discriminativeness vs. representativeness pattern of 30 horse gram genotypes in three different locations for the traits number of pods per plant (a), number of seeds per pod (b).

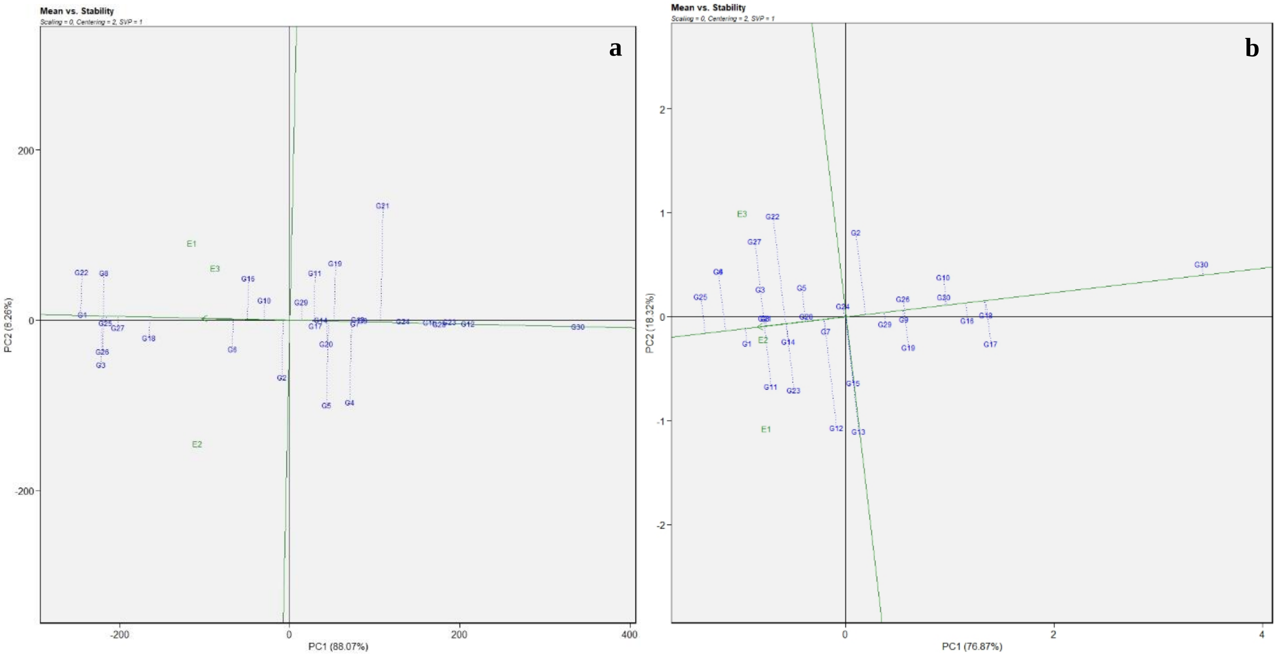

Supplementary Figure S5. Mean vs. stability plots for 30 horse gram genotypes in three different locations for the traits number of pods per plant (a), number of seeds per pod (b).

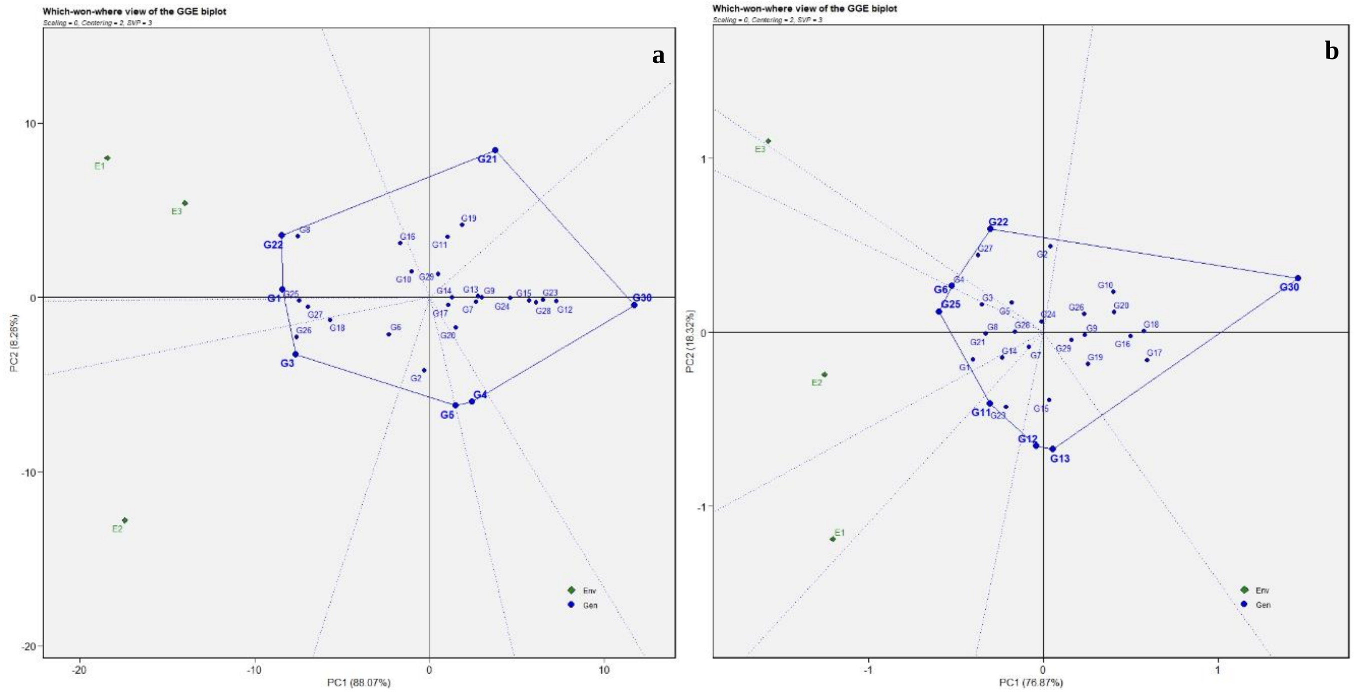

Supplementary Figure S6. “Which-won-where” plots for 30 horse gram genotypes in three different locations for the traits number of pods per plant (a), number of seeds per pod (b).

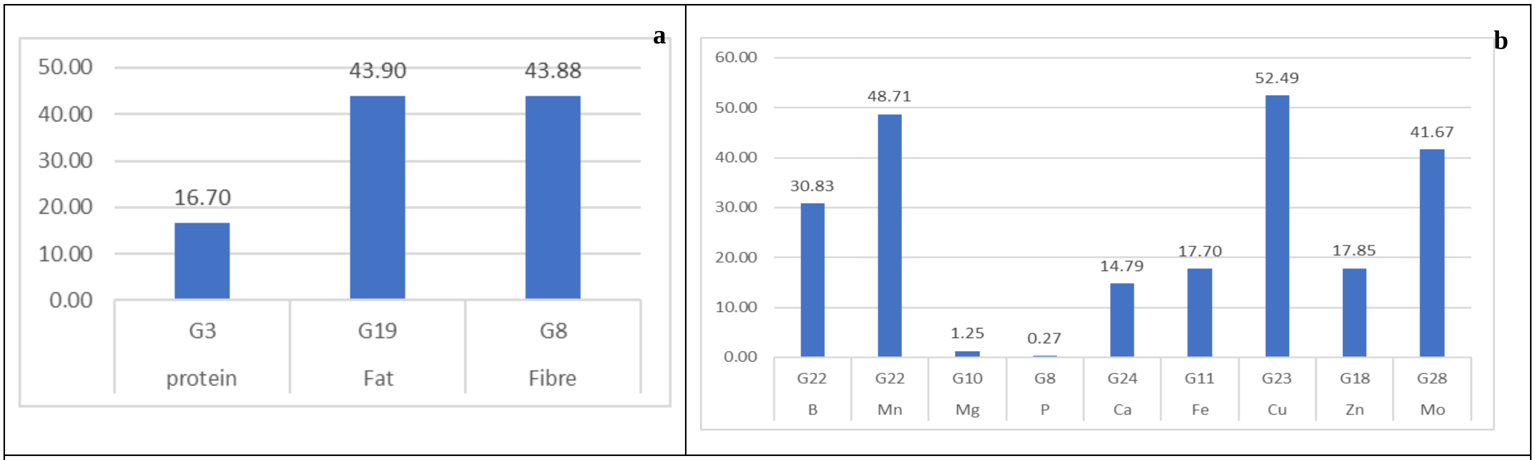

Supplementary Figure S7. a) Comparative supremacy of macronutrient-specific horse gram mutants for macronutrients over the parent PAIYUR 2 (in percentage). b) Comparative supremacy of micronutrient-specific horse gram mutants for micronutrients over the parent PAIYUR 2 (in percentage)

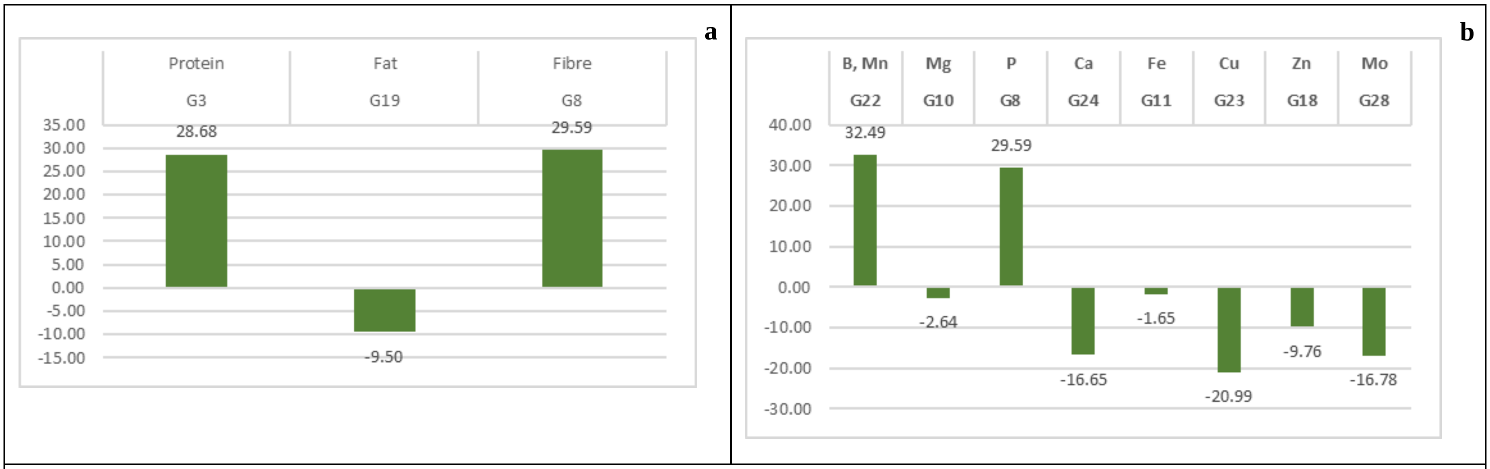

Supplementary Figure S8. a) Comparative seed yielding potential of macronutrient-specific horse gram mutants over the parent PAIYUR 2 (in percentage). b) Comparative seed yielding potential of micronutrient-specific horse gram mutants over the parent PAIYUR 2 (in percentage)

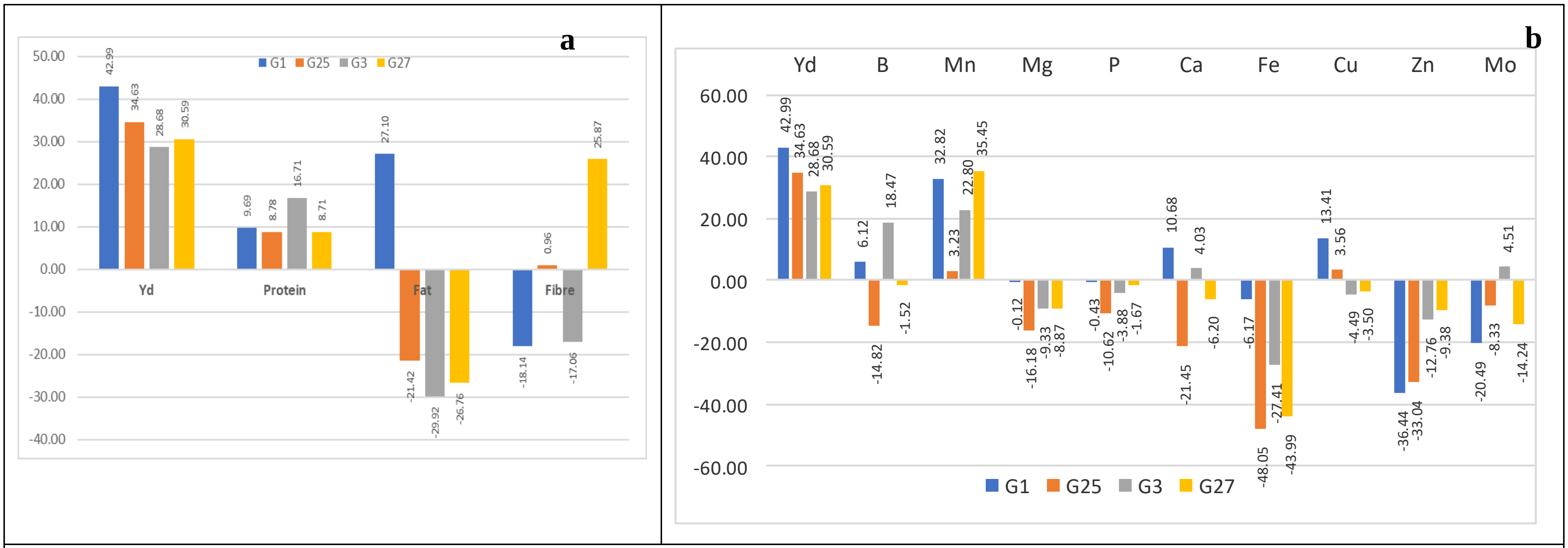

Supplementary Figure S9. a) Comparative seed yielding potential and macronutrient contents of identified stable and good-yielding horse gram mutants over the parent PAIYUR 2 (in %). b) Comparative seed yielding potential and micronutrient contents of identified stable and good-yielding horse gram mutants over the parent PAIYUR 2 (in %)

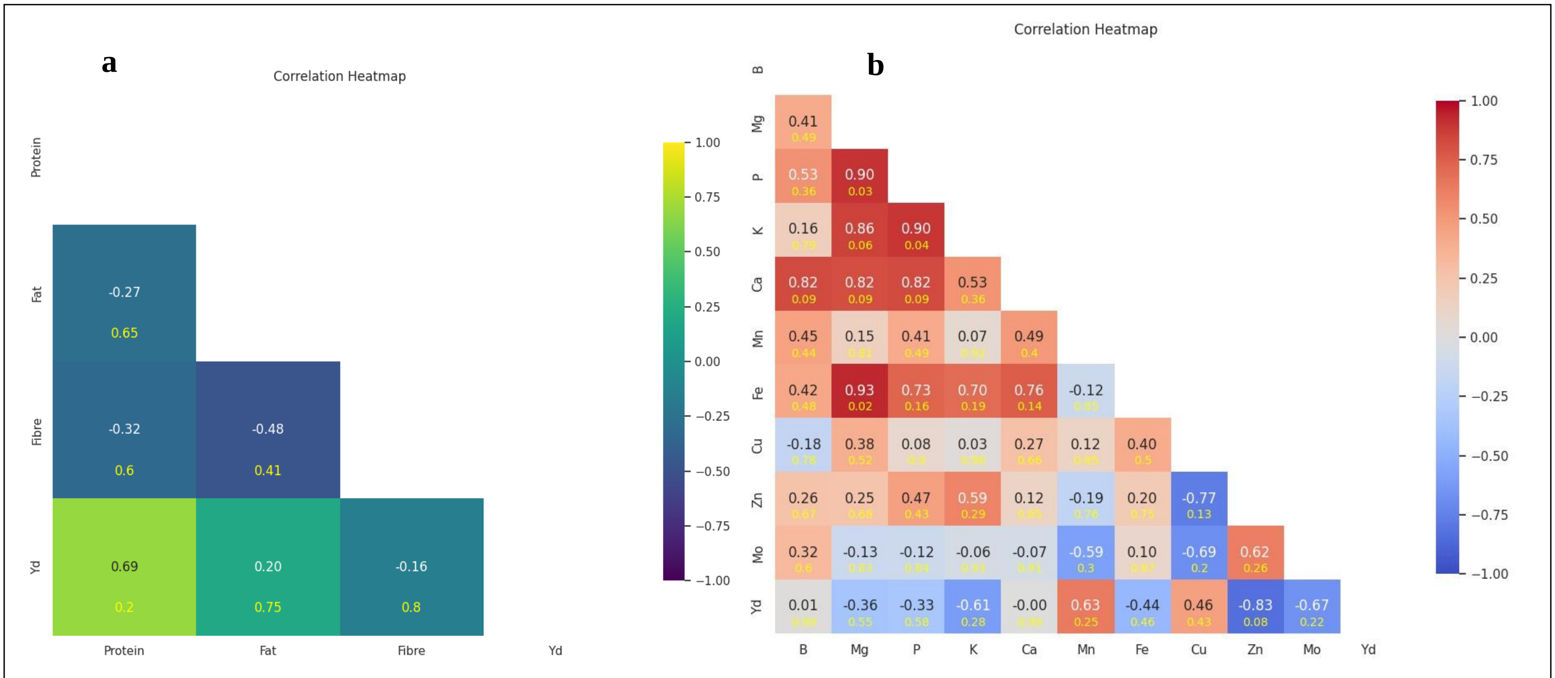

Supplementary Figure S10. a) Correlogram between good yielding and stable mutant's macronutrient content with their yield potential. b) Correlogram between good yielding and stable mutant's micronutrient content with their yield potential.

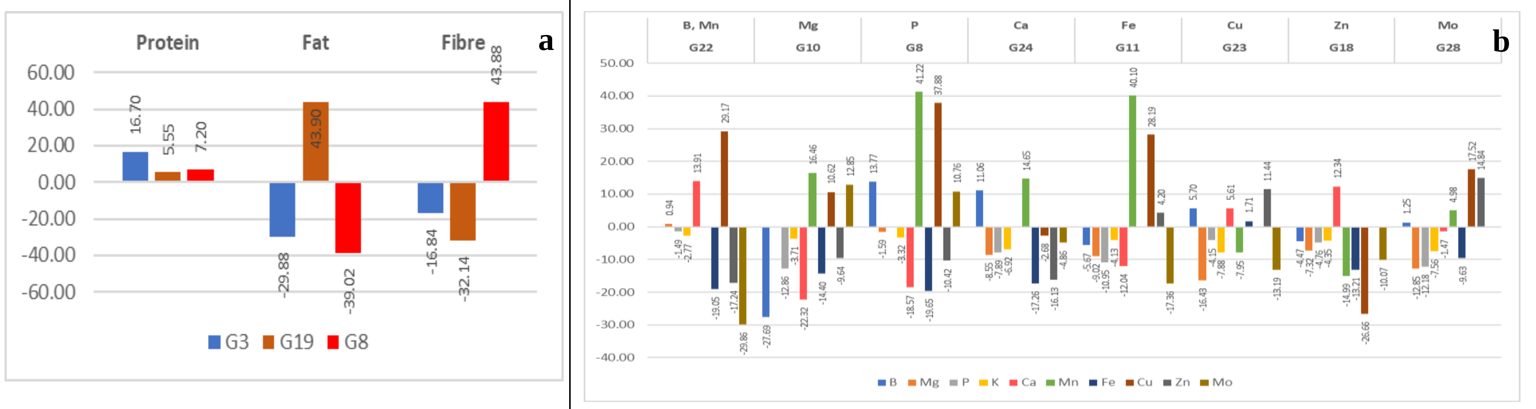

Supplementary Figure S11. a) Combined and comparative macronutrient supremacy of MaNSM over the parent PAIYUR 2. b) Combined and comparative micronutrient supremacy of MiNSM over the parent PAIYUR 2

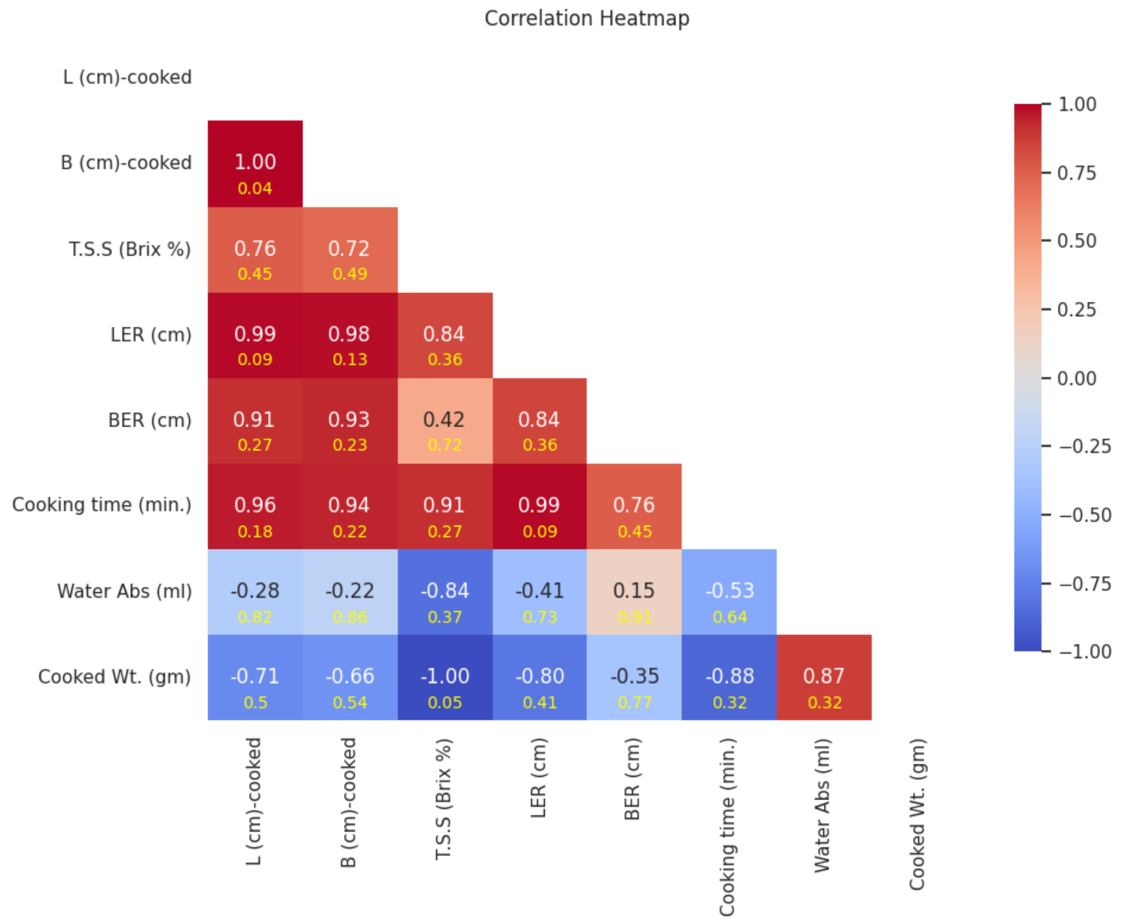

Supplementary Figure S12. Cooking quality parameters for the identified stable genotypes *viz.*, G1, G25 & PAIYUR 2

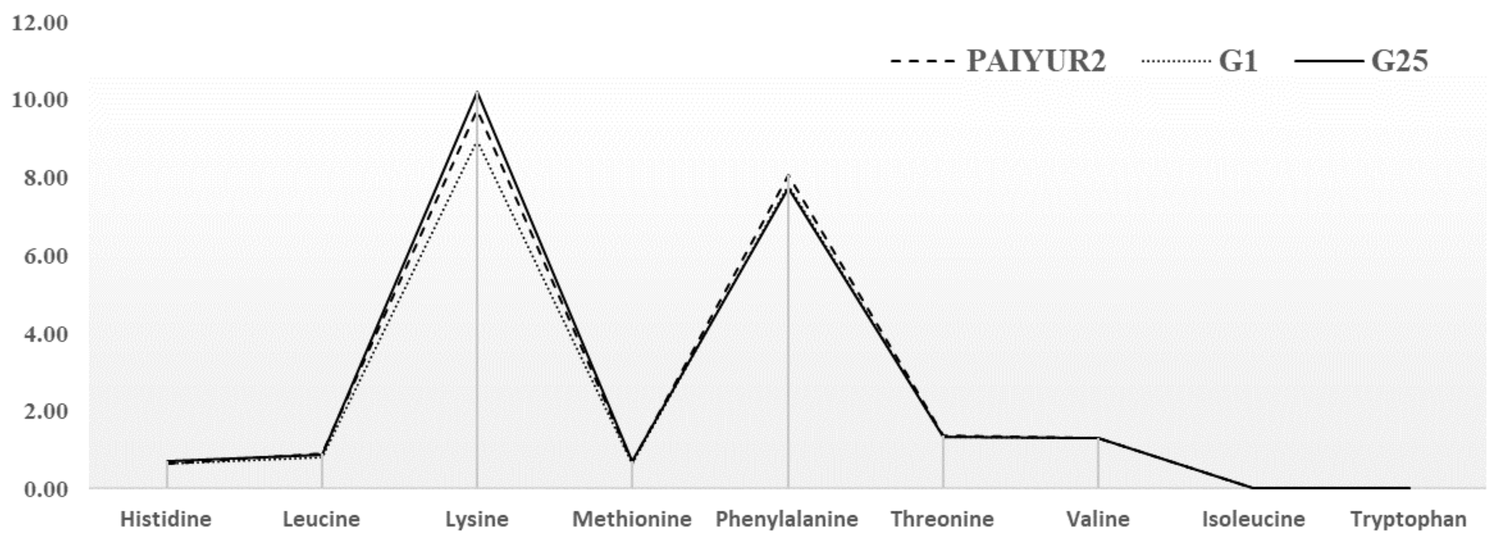

Supplementary Figure S13. Changes in the essential amino acids in comparison to total amino acids

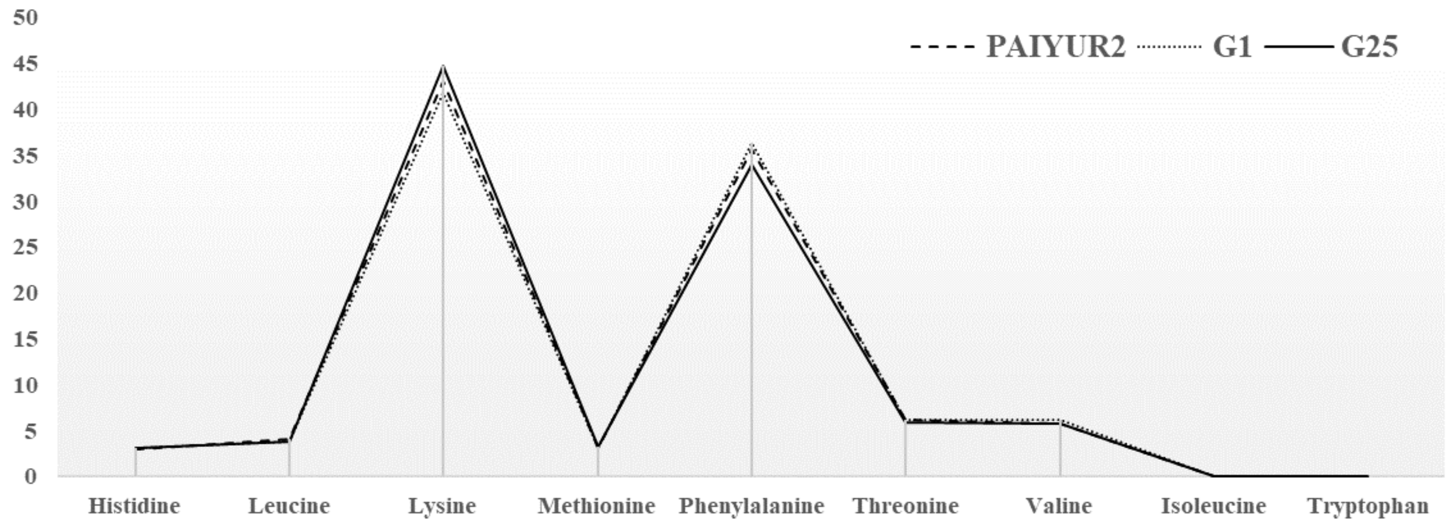

Supplementary Figure S14. Changes in the essential amino acids in comparison to total essential amino acids

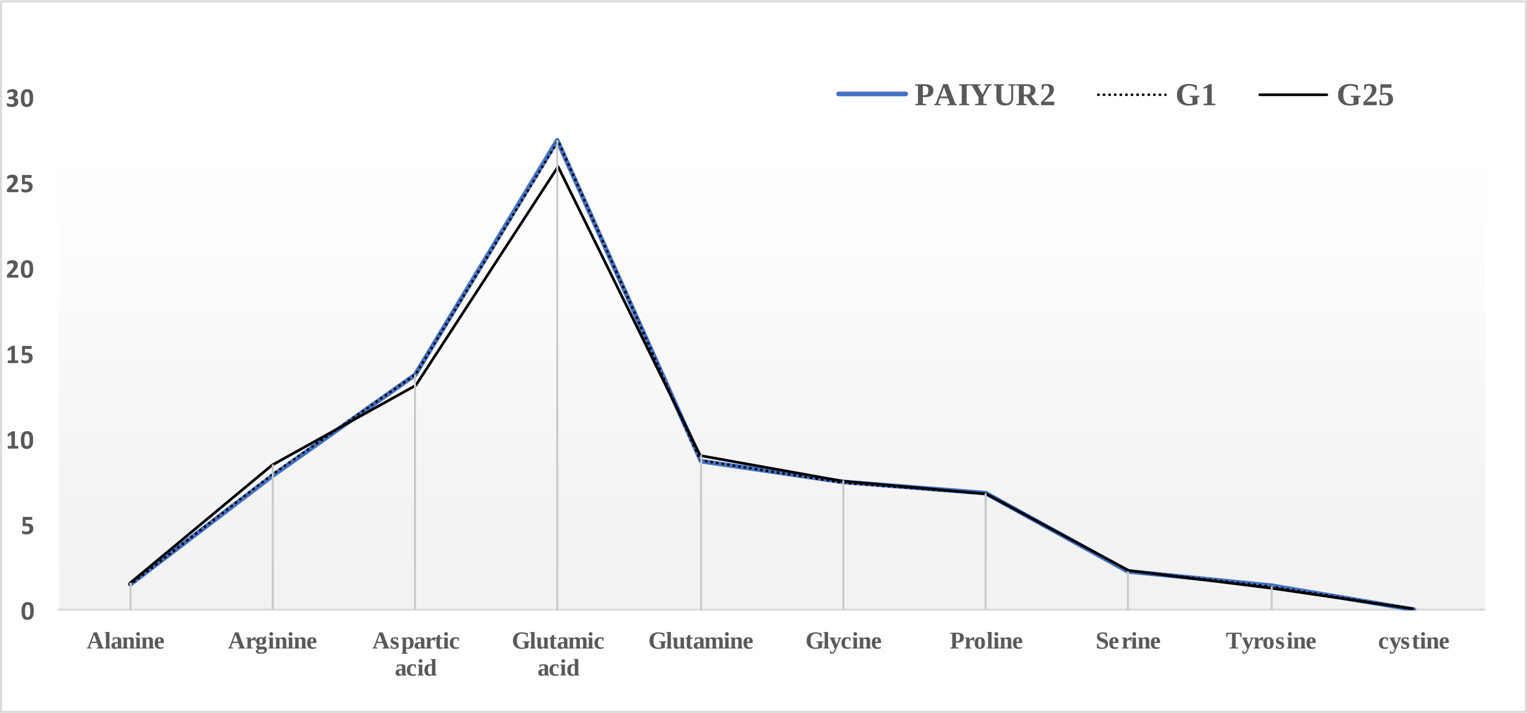

Supplementary Figure S15. Changes in the non-essential amino acids in comparison to total amino acids

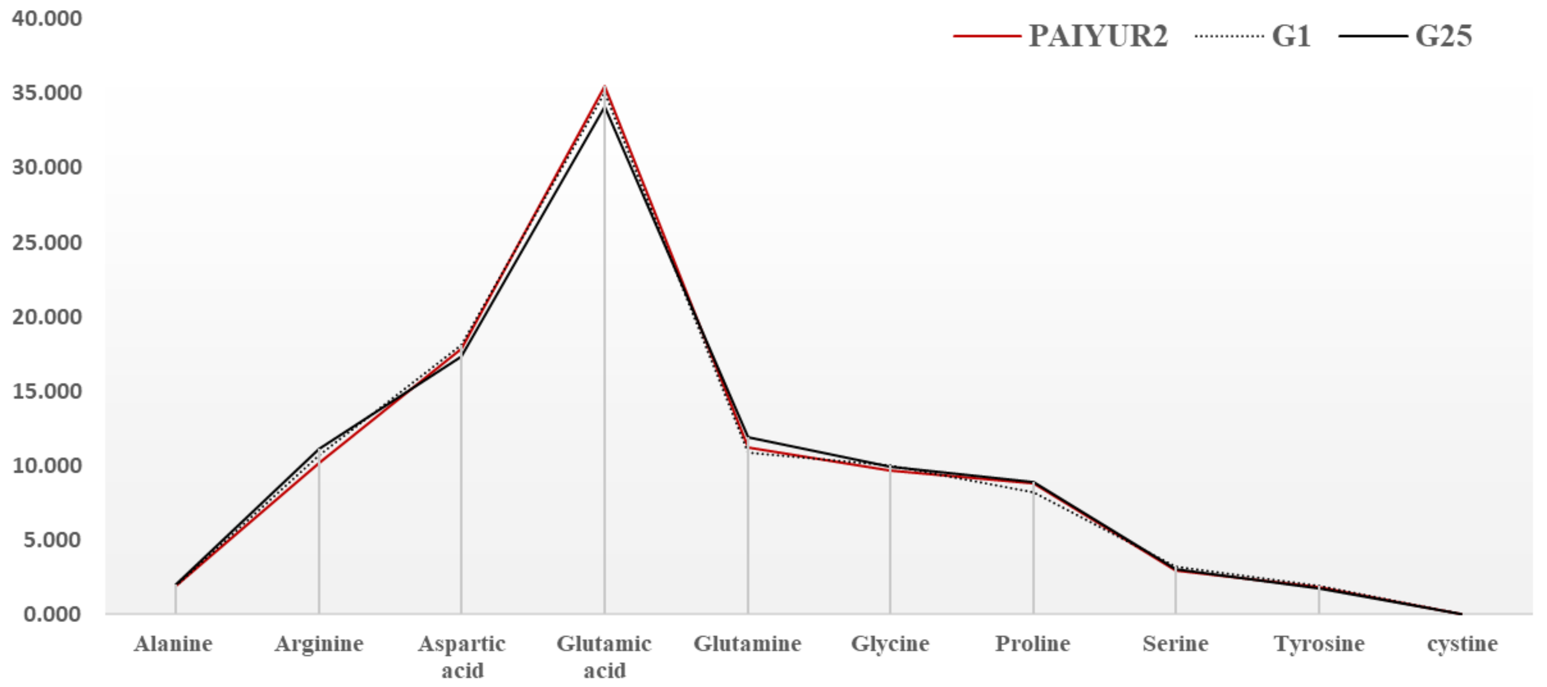

Supplementary Figure S16. Changes in the non-essential amino acids in comparison to total non-essential amino acids
